# Ultrasound screening for abdominal aortic aneurysm in high-risk women

**DOI:** 10.1093/bjs/znab220

**Published:** 2021-08-09

**Authors:** A Duncan, C Maslen, C Gibson, T Hartshorne, A Farooqi, A Saratzis, M J Bown

**Affiliations:** 1 Department of Cardiovascular Sciences & National Institute for Health Research (NIHR) Leicester Biomedical Research Centre (BRC), University of Leicester, Glenfield Hospital, Leicester, UK; 2 Leicester Vascular Institute, Glenfield Hospital, Leicester, UK; 3 Leicester City Clinical Commissioning Group, Leicester, UK

## Abstract

**Background:**

Population-wide ultrasound screening programmes for abdominal aortic aneurysm (AAA) for men have already been established in some countries. Women account for one third of aneurysm-related mortality and are four times more likely to experience an AAA rupture than men. Whole-population screening for AAA in women is unlikely to be clinically or economically effective. The aim of this study was to determine the outcomes of a targeted AAA screening programme for women at high risk of AAA.

**Method:**

Women aged 65–74 years deemed at high risk of having an AAA (current smokers, ex-smokers, or with a history of coronary artery disease) were invited to attend ultrasound screening (July 2016 to March 2019) for AAA in the Female Aneurysm screening STudy (FAST). Primary outcomes were attendance for screening and prevalence of AAA. Biometric data, medical history, quality of life (QoL) and aortic diameter on ultrasound imaging were recorded prospectively.

**Results:**

Some 6037 women were invited and 5200 attended screening (86.7 per cent). Fifteen AAAs larger than 29 mm were detected (prevalence 0.29 (95 per cent c.i. 0.18 to 0.48) per cent). Current smokers had the highest prevalence (0.83 (95 per cent c.i. 0.34 to 1.89) per cent) but lowest attendance (75.2 per cent). Three AAAs greater than 5.5 cm were identified and referred for consideration of surgical repair; one woman underwent repair. There was a significant reduction in patient-reported QoL scores following screening.

**Conclusion:**

A low prevalence of AAA was detected in high-risk women, with lowest screening uptake in those at highest risk. Screening for AAA in high-risk women may not be beneficial.

## Teaser

This a large empirical study of population screening for AAA in women with established cardiovascular risk factors. The key finding of this work is that screening high-risk women for AAA is not justified due to low disease prevalence. This is an important finding that will influence clinical practice, since current guidelines from the Society for Vascular Surgery, European Society for Vascular Surgery, and UK National Institute for Clinical Excellence all recommend screening for high-risk women.

## Introduction

Abdominal aortic aneurysm (AAA) is an important cardiovascular cause of death^1–3^. Aneurysms tend to remain symptom free with a long latent period^4^, but can rupture, which has a mortality rate greater than 80 per cent^5^. Aneurysms can be detected easily using ultrasound^6^. Population screening programmes have been introduced to screen men aged 65 for AAA by ultrasound in the UK, Sweden and Germany, and there is a selective screening programme in the USA. Screening has been shown to reduce aneurysm-related mortality by around 50 per cent and reduces all-cause mortality in those who attend for screening^7^. Population-wide screening for AAA in men across the UK is effective, providing the screening-detected prevalence of AAA remains above 0.35 per cent. Whole-population screening for AAA in women is unlikely to be clinically or economically effective^3^^,^^6^^,^^8^^,^^9^.

There is no high-quality randomized evidence investigating the effectiveness of AAA screening for women, and women are therefore not invited for screening^3^^,^^9–11^. This is supported by non-randomized data demonstrating lower disease prevalence in women than in men^3^^,^^9–13^. A recent modelling study has demonstrated that whole-population screening of women for AAA is unlikely to be clinically or economically effective; however, this was based on these historical estimates of prevalence and studies including all-comes (that is not populations at ‘high-risk’)^9^.

Women with AAA are currently four times more likely to experience an AAA rupture compared with men of the same age, have poorer outcomes after emergency surgery to repair an AAA and account for one-third of deaths due to ruptured AAA, despite a much lower prevalence reported in these historical studies^14^^,^^15^. In individuals affected, AAA represents a greater risk for women than for men. As a result, the question has arisen whether women are disadvantaged by the current screening models, which exclude them from established AAA screening programmes^3^^,^^9^^,^^11^.

Women with a history of smoking or coronary artery disease are more likely to have an AAA and these risk factors are consistently identified across studies^2^^,^^3^^,^^9–11^^,^^16^^,^^17^. Other identified risk factors for AAA in women include hyperlipidaemia and a family history of disease^18^. The main considerations with regard to extending AAA screening to women are the balance of the benefits and harms of screening, and cost-effectiveness. For a relatively rare disease such as AAA, the uptake of screening, disease prevalence and suitability of those women found with AAA for treatment will have a disproportionate effect on cost-effectiveness.

Whether the prevalence of AAA in high-risk groups is sufficient to consider selective screening for AAA, and whether high-risk women would attend for screening if invited is unknown.

The aim of the Female Aneurysm screening STudy (FAST) was to determine the uptake of screening and prevalence of AAA in a group of women deemed at high risk of having an AAA using data that were readily available in UK primary care records (white European ethnicity, current/ex-smokers and/or past history of coronary artery disease).

## Methods

### Study design and participants

The FAST was a cross-sectional study which invited women to attend screening clinics taking place either in their local primary care practice, or in hospital (secondary care). A population-screening approach was adopted with the exception that, for ethical reasons, women unlikely to benefit from screening (advanced dementia or palliative care) were excluded from invitation. Ethical approval was obtained from the East Midlands–Leicester South NHS Research Ethics Committee. The study protocol and data analysis plan were registered and published prior to recruitment at www.clinicaltrials.gov (registration number: NCT03277781); the study was funded by the National Institute for Health Research (NIHR) Research for Patient Benefit Programme (Reference PB-PG-0215–36027). The study complied with the Declaration of Helsinki and NHS Good Clinical Practice principles. Written informed consent was obtained from each participant.

Primary care records from 30 primary care (general practice) sites across Leicestershire and Northamptonshire were searched by a primary care practitioner at each primary care site to identify eligible women. The primary care team at each practice identified all eligible women at each practice based on the inclusion/exclusion criteria for the study. All eligible women at each participating practice were invited for screening.

Inclusion criteria were white women aged between 65 and 74 years with a current or ex-smoking habit, or a history of coronary artery disease. Smoking habit (current or ex-smoker) was based on each woman’s primary care record at the time of selection of the screening cohort. Coronary artery disease was defined as a primary care record indicating prior myocardial infarction, prior coronary artery bypass grafting or percutaneous coronary intervention, or angina. Exclusion criteria were a diagnosis of dementia or palliative care at the time of primary care record screening.

Between July 2016 and March 2019 (inclusive) women identified through this process were invited for screening. In order to obtain an unbiased estimate of attendance for screening, the invitation they received was an invitation to a pilot screening programme, not an invitation for research. This method was based upon previous screening uptake studies including the STRATEGIC cervical cancer screening trial and the ‘Lung Screen Uptake Trial’^19^^,^^20^. All women who attended were offered screening independent of their decision to participate in the research. All women attending for screening were offered screening, irrespective of whether they met the inclusion criteria for the study or not.

The study was conducted in two stages that used different invitation letters for screening. In both stages primary care sites identified the cohort of women to be invited for screening and mailed out a written invitation for screening. In the first state the invitation letter was modelled on the English NHS AAA Screening Programme (NAAASP) for men, whereby women were provided with a specific date and time for an appointment on their invitation letter. In the second stage, a ‘self-appointment’ model was used whereby the letter of invitation asked women to book an appointment by telephone if they wished to undergo screening. In keeping with NAAASP protocols that were in place at the time of study conduct, women who did not attend for screening or did not respond to an invitation to book a screening appointment were sent one further reminder invitation by post.

### Data collection

Additional data were collected from women who consented to participate in the research study. Demographic and clinical data were recorded at baseline, including medications, medical/surgical history and cardiovascular family history. Anthropometric data (height, weight, blood pressure and heart rate) were recorded using standard weighing scales and height gauges. Blood pressure was measured at a sitting position using a validated digital monitor (Omron M7, Omron Healthcare, Kyoto, Japan). The first 1000 participants completed a EQ-5D-5L quality-of-life (QoL) questionnaire immediately prior to their screening examination (baseline) and then via post after 6 months.

For each primary care practice involved in the study, the overall number of women invited and the proportion that attended were also recorded. Screening clinic utilization was calculated as the proportion of screening clinic appointments that were arranged where a woman attended for screening. Index of multiple deprivation score for each participant was obtained from primary care records where this was available.

### Screening protocol

Screening consisted of a single ultrasound scan of the abdominal aorta with images captured in two planes at the largest diameter identified. An AAA was defined as a maximal aortic size 3.0 cm or greater from inner edge to inner edge, as per established international guidance^21^. The full ultrasound protocol is detailed in the *Supplementary material*.

Following screening, there were five possible outcomes all of which were detailed in an outcome letter sent to the patient’s primary care practitioner. All aortas measuring 2.5 cm or greater were classified as ‘abnormal’ and those with measuring less than 2.5 cm as ‘normal’. An AAA was defined as 3.0 cm or greater. Women who screened normal were reassured and discharged. Those whose aorta could not be visualized were offered a second (repeat) scan at their primary care practice. If the aorta could still not be visualized they were referred to the University Hospitals of Leicester Vascular Studies Unit. Those with aortas between 2.5 cm and 2.9 cm were booked for a 5-year follow-up aortic ultrasound scan at their regional hospital vascular unit. The long-term clinical benefit of surveillance in this ‘sub-aneurysmal’ group is unknown but some studies have demonstrated a significant proportion will become aneurysmal within 5 years^22^. Women diagnosed with an AAA from 3.0–5.4 cm were offered cardiovascular risk factor modification advice, provided with an appointment with the local vascular unit for clinical assessment, and entered into follow-up with the local vascular surgery service. Surveillance scans were planned in accordance with NAAASP protocols for male AAA surveillance^23^.

Women with an AAA measuring 5.5 cm or greater were referred to their local vascular surgery unit for consideration of surgical repair.

### Statistical analysis and sample size calculations

The study sample size was set to determine an accurate estimate of AAA prevalence (primary outcome measure) in women with at least one risk factor for AAA (current smoker, ex-smoker or non-smoker with a history of coronary artery disease). Calculations using the Agresti–Couli method demonstrated that a sample size of 2626 women attending for screening would be adequate to determine AAA prevalence of 1 per cent (+/- 0.5 per cent with 99 per cent confidence)^24^. Power calculations determined that this sample size of 2626 would have excess power to test the hypothesis that AAA prevalence in the high-risk group was greater than the 0.35 per cent prevalence threshold of cost-effectiveness for AAA screening in men in the UK^8^. Overall, 1610 women would need to be screened to determine that AAA prevalence was greater than 0.35 per cent with 95 per cent power and 5 per cent significance. Based on an analysis of the shared clinical system used by primary care within the city of Leicester, and extrapolating this for the entire study area using Public Health Observatory data, the authors estimated there to be at least 8000 women aged 65–74 years currently smoking, 13 000 ex-smokers and 1700 non-smokers with coronary artery disease. They therefore aimed to invite 2626 smokers and 2626 ex-smokers. For non-smokers with coronary artery disease, the authors aimed to invite all 1700 but expected to screen up to 1400. Outcomes of interest were reported as proportions with 95 per cent confidence intervals. Continuous parametric variables were expressed as mean(s.d.); non-parametric variables were expressed as median (i.q.r.). Comparisons regarding attendance and prevalence between groups were performed using a χ^2^ test. Comparisons between continuous variables were performed using either a *t* test (parametric variables) or a Mann–Whitney U test (non-parametric variables); *P* < 0.050 was considered statistically significant. The data analysis plan and protocol were made available prior to commencing recruitment^25^^,^^26^.

## Results

Some 6037 women were invited for screening; 1762 were invited based on a primary care record indicating they were current smokers, 3709 as ex-smokers, and 527 non-smokers with a history of coronary artery disease. Thirty-nine women invited for screening were excluded from the study. Although the overall sample size was adequate to address the primary aim of the study at the completion of the funded recruitment period, subgroup sample sizes were inadequate for independent analysis at that time. The investigators made the decision to terminate recruitment due to futility of further recruitment as a result of unexpectedly low AAA prevalence (and therefore the likelihood that the harms of screening were greater than the benefit).

### Attendance for screening

Of the 5998 women invited for screening, 5200 (86.7 (95 per cent c.i. 85.8 to 87.5) per cent) attended their screening appointment and 5190 (86.5 (95 per cent c.i. 85.6 to 87.4) per cent) had ultrasound screening. Some 4613 women consented to collection of data for research (*Table 1*).

**Table 1:** Demographics of women attending for screening by invitational group

	All women	Invited as smokers	Invited as ex-smokers	Invited as non-smokers with history of CAD
**Number consented to extended data collection**	4613	1164	2996	453
**Mean age (years)**	69.6	69.3	69.6	70
**Mean height (cm)**	160	159.6	160.2	159.4
**Mean weight (kg)**	72.4	70.1	73.1	73.6
**Mean BMI (kg/m^2^)**	28.3	27.5	28.5	28.9
**Smoker**	726 (15.7)	654 (56.2)	65 (2.2)	7 (1.5)
**Ex-smoker**	3297 (71.5)	473 (40.6)	2719 (90.8)	105 (23.2)
**e-cigarette user**	248 (5.4)	143 (12.3)	101 (3.4)	4 (0.9)
**Diabetes**	452 (9.8)	122 (10.5)	274 (9.1)	56 (12.4)
**Stroke**	207 (4.5)	70 (6.0)	123 (4.1)	14 (3.1)
**Myocardial infarction**	205 (4.4)	43 (3.7)	90 (3.0)	72 (15.9)
**CABG**	40 (0.9)	14 (1.2)	10 (0.3)	16 (3.5)
**Coronary angiogram**	544 (11.8)	152 (13.1)	245 (8.2)	147 (32.5)
**Coronary stents**	171 (3.7)	34 (2.9)	71 (2.4)	66 (14.6)
**PAD**	68 (1.5)	40 (3.4)	26 (0.9)	2 (0.4)
**Hypertension**	2013 (43.6)	468 (40.2)	1287 (43.0)	258 (57.0)
**Antihypertensives**	1955 (42.4)	473 (40.6)	1233 (41.2)	249 (55.0)
**Hypercholesterolaemia**	2010 (43.6)	492 (42.3)	1257 (42.0)	261 (57.6)
**Aspirin**	537 (11.6)	133 (11.4)	273 (9.1)	131 (28.9)
**Clopidogrel**	167 (3.6)	61 (5.2)	92 (3.1)	14 (3.1)
**Warfarin**	107 (2.3)	29 (2.5)	62 (2.1)	16 (3.5)
**Statin**	1763 (38.2)	469 (40.3)	1060 (35.4)	234 (51.7)
**Anticoagulant**	117 (2.5)	25 (2.1)	77 (2.6)	15 (3.3)
**No medication**	2160 (46.8)	497 (42.7)	1533 (51.2)	130 (28.7)
**Family history of AAA**	325 (7.0)	83 (7.1)	217 (7.2)	25 (5.5)
Mother	103 (2.2)	30 (2.6)	62 (2.1)	11 (2.4)
Father	144 (3.1)	25 (2.1)	108 (3.6)	11 (2.4)
Brother	65 (1.4)	18 (1.5)	44 (1.5)	3 (0.7)
Sister	20 (0.4)	10 (0.9)	10 (0.3)	0 (0.0)
**Previous USS/CT/MRI**	2037 (44.2)	605 (52.0)	1192 (39.8)	240 (53.0)

Values in parentheses are the percentages of women with that finding within each invitational group. CAD, coronary artery disease; CABG, coronary artery bypass graft; PAD, peripheral artery disease; AAA, abdominal aortic aneurysm; USS, ultrasound scan.

**Fig. 1:**
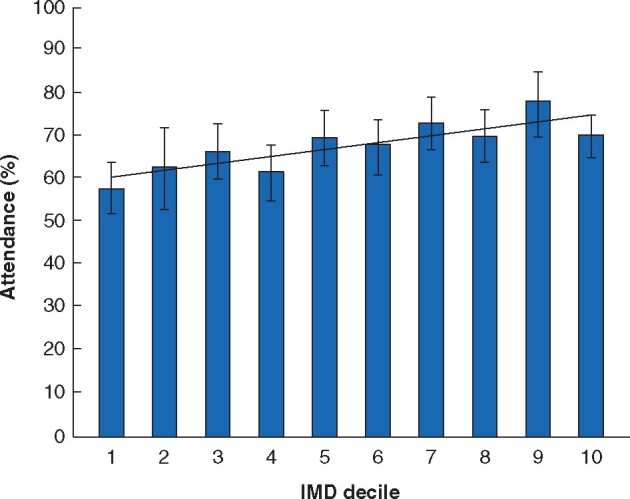
Attendance for abdominal aortic aneurysm screening by index of multiple deprivation decile Error bars represent 95 per cent confidence intervals. Lower index of multiple deprivation (IMD) decile (lower socioeconomic status) was associated with lower attendance for screening (*P* = 0.002, R^2^ = 0.7042)

Attendance for screening was lower in women with lower socioeconomic status based on index of multiple deprivation scores (R^2^ = 0.70, *P* = 0.002) (*Fig. 1*). Attendance differed by invitation group with significantly fewer of those invited as current smokers attending (75.1 per cent) compared with ex-smokers (91.3 per cent; *P* < 0.001) and non-smokers with a history of coronary artery disease (93.4 per cent; *P* < 0.001). There was no significant association between age and attendance.

Ten primary care sites submitted adequate data for the number of screening invitations sent out to be able to calculate clinic utilization accurately (3328 invitations in total). Overall attendance calculated from these data was 74.3 per cent. The authors compared attendance between those nine practices that had sent a pre-booked screening appointment with the invitation for screening (traditional invitation as used in the NAAASP programme for men, 2315 invitations) and the one large practice submitting data where women were asked to telephone the practice to make their own appointment (1015 invitations). Attendance was significantly better in the traditional invitation group (77.9 per cent) than the self-appointment group (62.7 per cent) (*P* = 0.001). Attendance by invitation group reflected the overall data with those invited as current smokers (67.7;0 per cent) when compared with those invited on the basis of coronary artery disease history (76.2 per cent) or ex-smokers (78.7 per cent). Traditional invitation was significantly better than self-appointment in all but the coronary artery disease group of patients. Clinic utilization (the proportion of clinic appointments set up that were attended) was significantly improved by using a self-appointment method with an increase in the proportion of patients taking up their first appointment from 65 to 98 per cent (33.4 (95 per cent c.i. 31.0 to 35.5) per cent increase, *P* = 0.001). In those practices using the traditional invitation model the clinic utilization by subgroup was as low as 54.1 per cent in current smokers, up to 67.7 per cent in participants with coronary artery disease only. For the practice using the self-appointment model, clinic utilization ranged from 92.6 to 100 per cent.

### Prevalence of AAA

Prevalence of AAA was 0.29 (95 per cent c.i. 0.18 to 0.48) per cent (15 AAA detected) in the 5169 women who attended for screening, were scanned and whose aorta could be visualized. Three of these 15 women had an AAA greater than 5.5 cm and were referred for consideration of surgical repair. Two were unfit for repair and one underwent successful endovascular AAA repair.

**Fig. 2:**
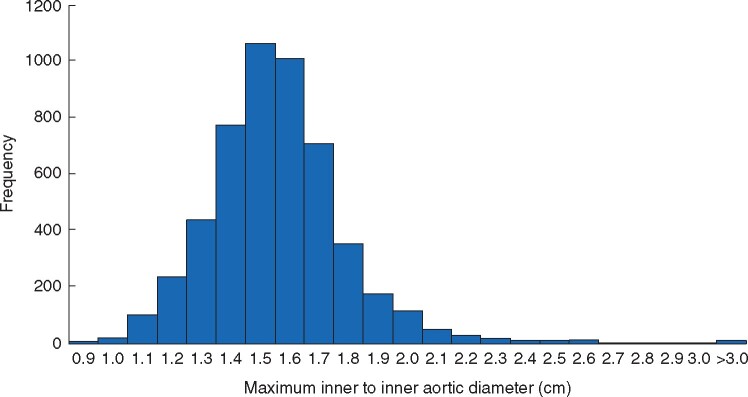
Distribution of aortic diameters in women aged 65–75 years invited with risk factors for abdominal aortic aneurysm Frequency of maximal aortic diameter in 5169 women who underwent scans. Range of diameters was from 0.8–6.0 cm with a mean(s.d.) aortic diameter of 1.57(0.27) cm. Fifteen women had an aortic diameter greater than 2.9 cm and were diagnosed with abdominal aortic aneurysm (AAA) (prevalence 0.29 per cent). Three of these 15 women had an AAA greater than 5.5 cm at screening and were referred for consideration of surgery

Inner-to-inner aortic diameter ranged from 0.8 cm to 6.0 cm with a mean(s.d.) aortic diameter of 1.57(0.27) cm (*Fig. 2*). Thirty-nine women (0.75 (95 per cent c.i. 0.54 to 1.04) per cent) were found to have sub-aneurysmal aortic dilatation: a maximal aortic diameter between 2.5 cm and 2.9 cm. AAA prevalence was highest in women invited on the basis of a primary care record indicating they were current smokers (0.69 (95 per cent c.i. 0.36 to 1.31) per cent). AAA prevalence in those invited as ex-smokers was 0.18 (95 per cent c.i. 0.08 to 0.39) per cent and 0 (95 per cent c.i. 0 to 0.77) per cent in non-smokers with coronary artery disease.

In those women that were screened who consented to data collection, analysis of AAA prevalence by actual smoking status recorded at the time of screening was possible (*Table 2*). Similar to the analyses based on primary care record smoking status, there was a higher prevalence of AAA in current smokers (0.83 (95 per cent c.i. 0.34 to 1.89) per cent) compared with ex-smokers (0.24 (95 per cent c.i. 0.11 to 0.49) per cent, *P* = 0.002). Combining both groups to consider ‘ever smokers’, the prevalence of AAA was 0.35 (95 per cent c.i. 0.2 to 0.6) per cent. The prevalence of AAA in women with coronary artery disease, independent of smoking status, was 0.52 (95 per cent c.i. 0.14 to 1.64) per cent.

**Table 2:** Prevalence of sub-aneurysmal aortic dilatation/abdominal aortic aneurysm by invitation group and directly recorded risk factors for abdominal aortic aneurysm

Invitation group/risk factor	**Normal aorta (aortic diameter <2.5** **cm)**	**Sub-aneurysmal aortic dilatation (aortic diameter 2.5–2.9** **cm)**	**AAA (aortic diameter ≥3.0** **cm)**
**All women**	98.96 (98.63–99.21)	0.75 (0.54–1.04)	0.29 (0.17–0.49)
**Invited as smoker**	97.78 (96.79–98.48)	1.53 (0.96–2.40)	0.69 (0.34–1.36)
**Invited as ex-smoker**	99.35 (99.00–99.58)	0.47 (0.28–0.78)	0.18 (0.07–4.10)
**Invited as non-smoker with CAD**	99.39 (98.22–99.79)	0.61 (0.21–1.78)	0.00 (0.00–0.77)
**Actual smoker**	96.96 (95.36–98.04)	2.21 (2.31–3.64)	0.83 (0.34–1.89)
**Actual ex-smoker**	99.18 (98.79–99.45)	0.58 (0.36–0.92)	0.24 (0.11–0.49)
**Actual non-smoker with CAD**	100.00 (96.67–100.00)	0.00 (0.00–2.67)	0.00 (0.00–2.67)
**Actual current smoker with CAD**	97.62 (91.73–99.35)	1.19 (0.21–6.44)	1.19 (0.21–6.44)
**Actual ex-smoker with CAD**	97.76 (95.46–98.95)	1.96 (0.86–4.17)	0.84 (0.29–2.44)

Values in parentheses are 95 per cent confidence intervals. AAA, abdominal aortic aneurysm; CAD, coronary artery disease.

Sub-aneurysmal aortas were also significantly more prevalent in current smokers (2.21 (95 per cent c.i. 1.31 to 3.64) per cent) than ex-smokers (0.58 (95 per cent c.i. 0.36 to 0.92) per cent) (*P* = 0.001). Three sub-aneurysmal aortas were recorded in the non-smoking group with coronary artery disease (0.6 (95 per cent c.i. 0.2 to 1.7) per cent).

### Quality of life

Quality of life was assessed using EQ-5D questionnaires. Analysis of these data revealed a significant decrease in quality of life in women screened for AAA between the time of screening and 6-month follow-up. Small numbers of data precluded meaningful analysis of quality-of-life data for women with AAA (3 participants with complete data) and women with sub-aneurysmal aortas (6 participants). Quality-of-life analyses are provided in the *Supplementary material* as are assessment of screening quality-assurance measures and an assessment of the accuracy of primary care records.

## Discussion

These results demonstrate that women at high risk of having an AAA will attend in relatively high numbers for AAA screening if invited using a process similar to that in existing AAA screening programmes for men. The observed uptake of AAA screening by women was similar to that for other screening programmes for women such as breast and cervical cancer screening programmes^27^. At the same time, AAA prevalence in women with risk factors for AAA was low at 0.29 per cent. This was lower than 0.35 per cent, the prevalence threshold below which AAA screening for men is likely to be ineffective. Whilst thresholds from cost-effectiveness models for men cannot be applied directly to women, when combined with recent modelling studies of whole-population screening for AAA in women that demonstrate low clinical and economic effectiveness^9^, these data suggest that AAA screening would be neither clinically nor economically effective in women at high risk of AAA. The authors did observe higher prevalence of AAA in women with a primary care record indicating they were current smokers (0.69 per cent).

The key strength of FAST is that it represents a large contemporary data set for prevalence of AAA in women at high risk of the disease, using a ‘real world’ design for invitation to a pilot screening programme. It also provides contemporary estimation for uptake of screening for AAA in high-risk women. Large data sets have been published consisting of self-appointment private screening clinics mainly from USA, however, these data are biased towards those who would present to screening, and does not represent a realistic view of a population-wide screening service^17^. The FAST findings demonstrate that the prevalence of AAA in women with risk factors for AAA is far lower than expected, in the population that attends for screening.

One limitation of this study is that, although a relatively large number of women were screened, the prevalence of AAA was much lower than expected, which limits the ability to draw conclusions beyond the overall prevalence in all high-risk women invited for screening. Prevalence data from past literature at the time of study design suggested a higher prevalence would be observed. This change in women is consistent with the decreasing prevalence of AAA seen in screening programmes for men^28^. The NAAASP is currently reporting a prevalence below that described in the historic trials which supported AAA screening in men^29^^,^^30^. Low AAA prevalence in women means that recruiting the sample sizes required for empirical studies to measure prevalence accurately is challenging and costly.

An inner to inner method to measure aortic diameter was used, consistent with the UK AAA screening programmes for men. This may have underestimated prevalence compared with other methods^31^ (outer to outer or leading edge to leading edge) since inner to inner measurements are approximately 2–3 mm less than these other methods^32^^,^^33^. If those aortae greater than 27 mm in diameter had been classified as AAA this would have increased the AAA prevalence to 0.54 (95 per cent c.i. 0.36 to 0.78) per cent in the entire study cohort (1.14 (95 per cent c.i. 0.64 to 1.88) per cent in smokers, 0.3 (0.14 to 0.54) per cent in ex-smokers and 0.61 (0.01 to 1.77) per cent in non-smoking women with CAD).

Irrespective of the method used to measure aortic diameter the threshold size at which to diagnose AAA in women remains under debate. Women have smaller aortas (mean diameter 1.57 cm in this study) than men (mean diameter 1.79 cm in NAAASP)^34^. Using a common size threshold for diagnosing AAA in women and men means that relative aortic diameter at this threshold compared to mean is greater in women than in men. Alternative approaches such as aortic size index (aortic diameter adjusted for body surface area) have been proposed^35^. A 3-cm threshold for diagnosis is approximately 1.9 times normal diameter in women. Since the definition of an aneurysm is based on dilation of 50 per cent or more above normal diameter^36^, the data add weight to the argument that the diagnostic threshold for AAA in women be revised downwards. Identifying the relationship between the method chosen to define an AAA and future clinical events is difficult. Obviously the best threshold to choose is one that reduces the risk of future aortic rupture by identifying all cases that would go on to rupture and minimizes harm from unnecessary surgical repairs. Designing a research study to define such a threshold is extremely challenging due to the low number of events when considering overall populations. It is more likely that this sort of information will be obtained from natural experiments and detailed epidemiological study of routine imaging data sets.

Screening women at high risk for AAA is unlikely to be either cost-effective or clinically effective if those AAA detected have a low likelihood of repair. In the large AAAs detected in FAST, only one of three was suitable for repair, bringing into question not only the clinical bearing, but also the ethical validity of screening for a disease which may not be treatable in the majority of cases. Despite this, the clinical relevance of detecting AAA in women will require a longer period of observation. In men the potential secondary benefits of identifying AAAs through improved cardiovascular risk management have been highlighted^37^^,^^38^. Whether women may also benefit from improved cardiovascular risk management by AAA detection in screening is unknown. These data do show limited statin/antithrombotic use in the high-risk women who attended for screening, suggesting the potential for additional benefit here. The low prevalence of AAA and the fact that these women were easily identified through electronic clinical records suggests that there are likely to be more efficient ways to identify not only the women with AAA, but a larger group of women who would benefit from improved cardiovascular risk management.

A reduction in quality-of-life outcomes was identified after screening. This suggests that a screening programme for AAA in women may cause harm. The quality-of-life data were limited by the lack of comparable data from an unscreened population and the inability to gather pre-screening quality-of-life data due to the study consent model. These potential harms of screening are an important area for future research.

The hypothesis of the study was that a targeted screening programme for women with risk factors for developing AAA would be an effective way of detecting AAA early in women and address the perceived disadvantage in a disease that remains highly morbid in the female population. However, although it remains the case that one-third of AAA deaths recorded in England are in women, screening between 65 and 74 years of age does not seem to be a clinically effective way of identifying AAA early in women. Longer-term follow-up of the cohort of 55 diseased aortas will help further to define the natural history of AAA in women, but in a rare disease that is becoming rarer it is becoming increasingly difficult to provide good-quality evidence for the best methods of screening and detection, let alone follow-up and treatment. A larger study would not be of benefit to calculate prevalence more accurately in what has been established as a rare disease (in women), even in those women deemed at high risk for developing it. Health services worldwide should exercise great caution before considering implementation of targeted screening of women for AAA.

## Supplementary Material

znab220_Supplementary_Data

## Notes

Data presented in this paper were presented in abstract form at the Vascular Society of Great Britain and Ireland scientific meeting, November 2019.
