# Supplementary material for: Ultrasound screening for abdominal aortic aneurysm in high-risk women
Source: Br J Surg. 2021 Aug 9;108(10):1192–8. doi: 10.1093/bjs/znab220 (PMC8545265; doi:10.1093/bjs/znab220)
Supplement: znab220_Supplementary_Data [file znab220_supplementary_data.zip › BOWn_FAST_manuscript_tables_and_figures_clean_final RJH edit 16.05.21.docx]

**Ultrasound screening for abdominal aortic aneurysm in high-risk women**

**Authors**

Mr Andrew Duncan MBBS BSc^1,2^;

Miss Caroline Maslen^1,2^;

Mrs Camilla Gibson^1^;

Mr Timothy Hartshorne^2^;

Professor Azhar Farooqi OBE FRCGP^3^;

Mr Athanasios Saratzis PhD FRCS^1,2^;

Professor Matthew J Bown MD FRCS^1,2^.

**Affiliations**

^1^Department of Cardiovascular Sciences & National Institute for Health Research (NIHR) Leicester Biomedical Research Centre (BRC), University of Leicester, Glenfield Hospital, Leicester, LE39QP

^2^Leicester Vascular Institute, Glenfield Hospital, Leicester, LE39QP

^3^Leicester City Clinical Commissioning Group, Leicester, LE38TB

**Short Title: The Female Aneurysm screening Study (FAST)**

**Corresponding Author**

**Mr A Saratzis,** Department of Cardiovascular Sciences & National Institute for Health Research (NIHR) Leicester Biomedical Research Centre (BRC), University of Leicester, Glenfield Hospital, Leicester, United Kingdom (UK), LE39QP**.**

E-mail: as875@le.ac.uk; Telephone: 0044 0116 252 3190.

**Funding**

This research was funded by the National Institute for Health Research (NIHR), Research for Patient Benefit (RfPB) Programme (Grant Reference Number PB-PG-0215-36027).

**Disclosures**

MJ Bown and A Saratzis are currently funded by the National Institute for Health Research (NIHR; References: NIHR202008, NIHR130075, NIHR200601, NIHR300059) and the British Hearth Foundation (BHF; Reference: RG/18/10/33842) for research relating to abdominal aortic aneurysms. A Saratzis is currently funded by Abbott Medical Ltd. (no reference) for research relating to peripheral arterial disease and receives honoraria from Philips Healthcare for research relating to abdominal aortic aneurysm treatment(s).

**Word count: 5,151**

**Original article (observational study)**

**Study Registration:** The study protocol and data analysis plan were registered and published prior to recruitment at clinicaltrials.gov (NCT03277781).

Data presented in this paper was presented in abstract form at the Vascular Society of Great Britain and Ireland scientific meeting November 2019.

**ABSTRACT**

**Background:** Population-wide ultrasound screening programmes for abdominal aortic aneurysm (AAA) for men have already been established in some countries. Women account for one third of aneurysm-related mortality and are four times more likely to experience an AAA rupture than men. Whole population screening for AAA in women is unlikely to be clinically or economically effective. The aim of this study was to determine the outcomes of a targeted AAA screening programme for women at high risk of AAA.

**Method:** Women aged 65 to 74 years deemed at high risk of having an AAA (current smokers, ex-smokers, or with a history of coronary artery disease) were invited to attend ultrasound screening (July 2016 to March 2019) for AAA in the Female Aneurysm screening STudy (FAST). Primary outcomes were attendance for screening and prevalence of AAA. Biometric data, medical history, Quality-of-Life (QoL), and aortic diameter on ultrasound imaging were prospectively recorded.

**Results:** Some 6,037 women were invited and 5,200 attended screening (86·7%); Fifteen AAAs >29mm were detected [prevalence 0·29%, 95% Confidence Interval (CI) 0·18% - 0·48%)]. Current smokers had the highest prevalence (0·83%, 95% CI: 0·34% - 1·89%) but lowest attendance (75·2%). Three AAAs greater than 5·5cm were identified and referred for consideration of surgical repair; one woman underwent repair. There was a significant reduction in patient-reported QoL scores following screening.

**Conclusion:** A low prevalence of AAA was detected in high-risk women, with lowest screening uptake in those at highest risk. Screening for AAA in high-risk women may not be beneficial.

TOC summary

This a large empirical study of population screening for AAA in women with established cardiovascular risk factors. The key finding of our work is that screening high-risk women for AAA is not justified due to low disease prevalence. This is an important finding that will influence clinical practice since current guidelines from the Society for Vascular Surgery, European Society for Vascular Surgery, and UK National Institute for Clinical Excellence all recommend screening for high-risk women.

**INTRODUCTION**

Abdominal aortic aneurysm (AAA) is an important cardiovascular cause of death.^1-3^ Aneurysms tend to remain symptom free with a long latent period,^4^ but can rupture, which has a mortality rate greater than 80%.^5^ Aneurysms can easily be detected using ultrasound.^6^ Population screening programmes have been introduced to screen men aged 65 for AAA by ultrasound in the UK, Sweden, and Germany, and there is a selective screening programme in the USA. Screening has been shown to reduce aneurysm-related mortality by around 50% and reduces all-cause mortality in those who attend for screening.^7^ Population-wide screening for AAA in men across the UK is effective, providing the screen-detected prevalence of AAA remains above 0·35%. Whole population screening for AAA in women is unlikely to be clinically or economically effective.^3, 6, 8, 9^

There is no high-quality randomised evidence investigating the effectiveness of AAA screening for women, and women are therefore not invited for screening.^3, 9-11^ This is supported by non-randomised data demonstrating lower disease prevalence in women than in men. ^3, 9-13^ A recent modelling study has demonstrated that whole population screening of women for AAA is unlikely to be clinically or economically effective; however, this was based on these historical estimates of prevalence and studies including all-comes (i.e. not populations at “high-risk”).^9^

Women with AAA are currently four times more likely to experience an AAA rupture compared to men of the same age, have poorer outcomes after emergency surgery to repair an AAA, and account for one-third of deaths due to ruptured AAA, despite a much lower prevalence reported in these historical studies.^16, 17^ In individuals affected, AAA represents a greater risk for women than men. As a result, the question has arisen whether women are disadvantaged by the current screening models, which exclude them from established AAA screening programmes.^3, 9, 11^

Women with a history of smoking or coronary artery disease are more likely to have an AAA and these risk factors are consistently identified across studies.^2, 3, 9-11, 15, 18^ Other identified risk factors for AAA in women include hyperlipidaemia and a family history of disease.^19^ The main considerations with regards to extending AAA screening to women are the balance of the benefits and harms of screening, and cost-effectiveness. For a relatively rare disease such as AAA, the uptake of screening, disease prevalence and suitability of those women found with AAA for treatment will have a disproportionate effect on cost-effectiveness.

Whether the prevalence of AAA in high risk groups is sufficient to consider selective screening for AAA, and whether high risk women would attend for screening if invited is unknown.

The aim of the Female Aneurysm screening STudy (FAST) was to determine the uptake of screening and prevalence of AAA in a group of women deemed at high risk of having an AAA using data that was readily available in UK primary care records (white European ethnicity, current/ex-smokers and/or past history of coronary artery disease).

**METHODS**

**Study design and participants**

The FAST Study was a cross sectional study which invited women to attend screening clinics taking place either in their local primary care practice, or in hospital (secondary care). A population screening approach was adopted with the exception that, for ethical reasons, women unlikely to benefit from screening (advanced dementia or palliative care) were excluded from invitation. Ethical approval was obtained from the East Midlands – Leicester South NHS Research Ethics Committee (REC). The study protocol and data analysis plan were registered and published prior to recruitment at clinicaltrials.gov (NCT03277781); the study was funded by the National Institute for Health Research (NIHR) Research for Patient Benefit Programme (Reference PB-PG-0215-36027). The study complied with the Declaration of Helsinki and NHS Good Clinical Practice (GCP) principles. Written informed consent was obtained from each participant.

Primary care records from thirty primary care (general practice) sites across Leicestershire and Northamptonshire were searched by a primary care practitioner at each primary care site to identify eligible women. The primary care team at each practice identified all eligible women at each practice based on the inclusion/exclusion criteria for the study. All eligible women at each participating practice were invited for screening.

***Inclusion criteria***

White women aged between 65 and 74 with a current or ex-smoking habit, or a history of coronary artery disease. Smoking habit (current or ex-smoker) was based on each woman’s primary care record at the time of selection of the screening cohort. Coronary artery disease was defined as a primary care record indicating prior myocardial infarction, prior coronary artery bypass grafting or percutaneous coronary intervention, or angina.

***Exclusion criteria***

A diagnosis of dementia or palliative care at the time of primary care record screening.

Between July 2016 and March 2019 (inclusive) women identified through this process were invited for screening. In order to obtain an unbiased estimate of attendance for screening, the invitation they received was an invitation to a pilot screening programme, not an invitation for research. This method was based upon previous screening uptake studies including the STRATEGIC cervical cancer screening trial and the “Lung Screen Uptake Trial”.^20, 21^ All women who attended were offered screening independent of their decision to participate in the research. All women attending for screening were offered screening, irrespective of whether they met the inclusion criteria for the study or not.

The study was conducted in two stages that used different invitation letters for screening. In both stages primary care sites identified the cohort of women to be invited for screening and mailed out a written invitation for screening. In the first state the invitation letter was modelled on the English NHS AAA Screening Programme (NAAASP) for men, whereby women were provided with as specific a date and time for an appointment on their invitation letter. In the second stage, a “self-appointment” model was used whereby the letter of invitation asked women to book an appointment by telephone if they wished to undergo screening. In keeping with NAAASP protocols that were in place at the time of study conduct, women who did not attend for screening or did not respond to an invitation to book a screening appointment were sent one further reminder invitation by post.

**Data collection**

Additional data was collected from women who consented to participate in the research study. Demographic and clinical data were recorded at baseline, including medications, medical / surgical history, and cardiovascular family history. Anthropometric data (height, weight, blood pressure, and heart rate) were recorded using standard weighing scales and height gauges. Blood pressure was measured at a sitting position using a validated digital monitor (Omron M7, Omron Healthcare, Kyoto, Japan). The first 1,000 participants completed a EQ-5D-5L Quality-of-Life (QoL) questionnaire immediately prior to their screening examination (baseline) and then via post after six months.

For each primary care practice involved in the study, the overall number of women invited and the proportion that attended was also recorded. Screening clinic utilisation was calculated as the proportion of screening clinic appointments that were arranged where a woman attended for screening. Index of multiple deprivation score for each participant was obtained from primary care records where this was available.

**Screening protocol**

Screening consisted of a single ultrasound scan of the abdominal aorta with images captured in two planes at the largest diameter identified. An AAA was defined as a maximal aortic size ≥3·0cm from inner edge to inner edge, as per established international guidance.^22^ The full ultrasound protocol is detailed in the Supplementary information.

Following screening, there were five possible outcomes all of which were detailed in an outcome letter sent to the patient’s primary care practitioner. All aortas measuring ≥2·5cm were classified as “abnormal” and those with measuring <2·5cm as “normal”. An AAA was defined as ≥3·0cm. Women who screened normal were reassured and discharged. Those whose aorta could not be visualised, were offered a second (repeat) scan at their primary care practice. If the aorta could still not be visualised they were referred to the University Hospitals of Leicester Vascular Studies Unit. Those with aortas between 2·5cm and 2·9cm were booked for a five-year follow up aortic ultrasound at their regional hospital vascular unit. The long-term clinical benefit of surveillance in this ‘sub-aneurysmal’ group is unknown but some studies have demonstrated a significant proportion will become aneurysmal within five years.^23^ Women diagnosed with an AAA from 3·0cm to 5·4cm were offered cardiovascular risk factor modification advice, provided with an appointment with the local vascular unit for clinical assessment, and entered into follow up with the local vascular surgery service. Surveillance scans were planned in accordance to NAAASP protocols for male AAA surveillance.^24^

Women with an AAA measuring ≥5·5cm were referred to their local vascular surgery unit for consideration of surgical repair.

**Statistical analysis and sample size calculations**

The study sample size was set to determine an accurate estimate of AAA prevalence (primary outcome measure) in women with at least one risk factor for AAA (current smoker, ex-smoker or non-smoker with a history of coronary artery disease). Calculations using the Agresti-Couli method demonstrated that a sample size of 2,626 women attending for screening would be adequate to determine AAA prevalence of 1% (+/- 0·5% with 99% confidence).^25^ Power calculations determined that this sample size of 2,626 would have excess power to test the hypothesis that AAA prevalence in our high-risk group was greater than the 0·35% prevalence threshold of cost-effectiveness for AAA screening in men in the UK.^8^ Overall, 1,610 women would need to be screened to determine that an AAA prevalence of 1% was greater than 0·35% with 95% power and 5% significance. Based on an analysis of the shared clinical system used by primary care within the city of Leicester, and extrapolating this for the entire study area using Publish Health Observatory data, we estimated there to be at least 8,000 women aged 65 to 74 currently smoking, 13,000 ex-smokers and 1,700 non-smokers with coronary artery disease. We therefore aimed to invite 2,626 smokers and 2,626 ex-smokers. For non-smokers with coronary artery disease, we aimed to invite all 1,700 but expected to screen up to 1,400. Outcomes of interest were reported as proportions with a 95% Confidence Interval (CI). Continuous parametric variables were expressed as mean values and Standard Deviation (SD); non-parametric variables were expressed as median values and inter-quartile ranges (IQRs). Comparisons regarding attendance and prevalence between groups were performed using a chi-square test. Comparisons between continuous variables were performed using either a t-test (parametric variables) or a Mann-Whitney U test (non-parametric variables); a p value <0·05 was considered statistically significant. The data analysis plan and protocol were made available prior to commencing recruitment^.26, 27^

**Role of the funding source**

The funder of the study had no role in study design, data collection, data analysis, data interpretation, or writing of the manuscript. AD, MJB, and AS had full access to all the data in the study and had final responsibility for the decision to submit for publication.

**RESULTS**

Some 6,037 women were invited for screening; 1,762 were invited based on a primary care record indicating they were current smokers, 3,709 as ex-smokers, and 527 non-smokers with a history of coronary artery disease. 39 women invited for screening were excluded from the study. Whilst the overall sample size was adequate to address the primary aim of the study at the completion of the funded recruitment period, sub-group sample sizes were inadequate for independent analysis at that time. The investigators made the decision to terminate recruitment due to futility of further recruitment as a result of unexpectedly low AAA prevalence (and therefore the likelihood that the harms of screening were greater than the benefit).

**Attendance for screening**

Of the 5,998 women invited for screening, 5,200 (86·7%; 95% CI: 85·8% - 87·5%) attended their screening appointment and 5,190 (86·5%; 95% CI: 85·6% - 87·4%) had ultrasound screening. 4613 women consented to collection of data for research (Table 1).

Attendance for screening was lower in women with lower socio-economic status based on index of multiple deprivation scores (R^2^=0·70, p=0·002) (Figure 1). Attendance differed by invitation group with significantly fewer of those invited as current smokers attending (75·1%) compared to ex-smokers (91·3%; p=<0·0002) and non-smokers with a history of coronary artery disease (93·4%; p=<0·0002). There was no significant association between age and attendance.

Ten primary care sites submitted adequate data for the number of screening invitations sent out to be able to accurately calculate clinic utilisation (3,328 invitations in total). Overall attendance calculated from this data was 74·3%. We compared attendance between those nine practices that had sent a pre-booked screening appointment with the invitation for screening (traditional invitation as used in the NAAASP programme for men, 2315 invitations) and the one large practice submitting data where women were asked to telephone the practice to make their own appointment (1,015 invitations). Attendance was significantly better in the traditional invitation group (77·9%) than the self-appointment group (62·7%) (p=0·0001). Attendance by invitation group reflected the overall data with those invited as current smokers (67·0%) when compared to those invited on the basis of coronary artery disease history (76·2%) or ex-smokers (78·7%). Traditional invitation was significantly better than self-appointment in all but the CAD group of patients. Clinic utilisation (the proportion of clinic appointments set up that were attended) was significantly improved by using a self-appointment method with an increase in the proportion of patients taking up their first appointment from 65% to 98% (33·4% increase (95% CI: 31·0% - 35·5%, p=0·0001). In those practices using the traditional invitation model the clinic utilisation by sub-group was as low as 54·1% in current smokers, up to 67·7% in participants with coronary artery disease only. For the practice using the self-appointment model, clinic utilisation ranged from 92·6% to 100%.

**Prevalence of AAA**

Prevalence of AAA was 0·29% (15 AAA detected, 95% CI: 0·18% - 0·48%) in the 5169 women who attended for screening, were scanned and the aorta could be visualised. Three of these 15 women had an AAA greater than 5.5cm and were referred for consideration of surgical repair. Two were unfit for repair and one underwent successful endovascular AAA repair.

Inner-to-inner aortic diameter ranged from 0·8cm to 6·0cm with a mean aortic diameter of 1·57cm (SD=0·27cm) (Figure 2). Thirty-nine women (0·75%) were found to have sub-aneurysmal aortic dilatation i.e. a maximal aortic diameter between 2·5cm and 2·9cm (95% CI: 0·54% – 1·04%). AAA prevalence was highest in women invited on the basis of a primary care record indicating they were current smokers (0·69%; 95% CI: 0·36% - 1·31%). AAA prevalence in those invited as ex-smokers was 0·18% (95% CI: 0·08% - 0·39%) and 0% in non-smokers with coronary artery disease (95% CI: 0 - 0·77%).

In those women that were screened who consented to data collection, analysis of AAA prevalence by actual smoking status recorded at the time of screening was possible (Table 2). Similar to the analyses based on primary care record smoking status there was a higher prevalence of AAA in current smokers (0·83%, 95% CI: 0·34% - 1·89%) compared to ex-smokers (0·24%, 95% CI: 0·11% - 0·49%) (p=0·02). Combining both groups to consider ‘ever smokers’, the prevalence of AAA was 0·35% (95% CI: 0·2% - 0·6%). The prevalence of AAA in women with coronary artery disease, independent of smoking status, was 0·52% (95% CI: 0·14 - 1·64%).

Sub-aneurysmal aortas were also significantly more prevalent in current smokers (2·21%, 95% CI: 1·31% - 3·64%) than ex-smokers (0·58%, 95% CI: 0·36% - 0·92%) (p=0·0001). Three sub-aneurysmal aortas were recorded in the non-smoking group with coronary artery disease (0·6%, 95% CI: 0·2% - 1·7%).

**Quality of life**

Quality of life was assessed using EQ5D questionnaires. Analysis of these data revealed a significant decrease in quality of life in women screened for AAA between the time of screening and six-month follow-up. Small numbers of data precluded meaningful analysis of quality of life data for women with AAA (n=3 with complete data) and women with sub-aneurysmal aortae (n=6). Quality of life analyses are provided in the Supplementary information as are assessment of screening quality assurance measures and an assessment of the accuracy of primary care records.

**DISCUSSION**

These results demonstrate that women at high risk of having an AAA will attend in relatively high numbers for AAA screening if invited using a process similar to that in existing AAA screening programmes for men. The observed uptake of AAA screening by women was similar to other screening programmes for women such as breast and cervical cancer screening programmes.^28^ At the same time, AAA prevalence in women with risk factors for AAA was low at 0·29%. This was lower than 0·35%, the prevalence threshold below which AAA screening for men is likely to be ineffective. Whilst thresholds from cost-effectiveness models for men cannot be applied directly to women, when combined with recent modelling studies of whole population screening for AAA in women that demonstrate low clinical and economic effectiveness,^9^ these data suggest that AAA screening would be neither clinically nor economically effective in women at high risk of AAA. We did observe higher prevalence of AAA in women with a primary care record indicating they were current smokers (0.69%).

The key strength of FAST is that it represents a large contemporary data set for prevalence of AAA in women at high risk of the disease, using a ‘real world’ design for invitation to a pilot screening programme. It also provides contemporary estimation for uptake of screening for AAA in high risk women. Large datasets have been published consisting of self-appointment private screening clinics mainly from America, however, this data is biased towards those who would present to screening, and does not represent a realistic view of a population-wide screening service.^18^ The FAST findings demonstrate that the prevalence of AAA in women with risk factors for AAA is far lower than expected, in the population that attends for screening.

One limitation of this study is that whilst a relatively large number of women were screened, the prevalence of AAA that was much lower than expected which limits the ability to draw conclusions beyond the overall prevalence in all high-risk women invited for screening. Prevalence data from past literature at the time of study design suggested a higher prevalence would be observed. This change in women is consistent with the decreasing prevalence of AAA seen in screening programmes for men.^29^ The NAAASP is currently reporting a prevalence below that described in the historic trials which supported AAA screening in men.^14, 30^ Low AAA prevalence in women means that recruiting the sample sizes required for empirical studies to accurately measure prevalence is challenging and costly.

An inner to inner method to measure aortic diameter was used, consistent with the UK AAA screening programmes for men. This may have under-estimated prevalence compared to other methods^31^ (outer to outer or leading edge to leading edge) since inner to inner measurements are approximately 2-3mm less than these other methods.^32^ ^33^ If those aortae greater than 27mm in diameter had been classified as AAA this would have increased the AAA prevalence to 0.54% (95%CI 0.36% to 0.78%) in the entire study cohort (1.14% (0.64% to 1.88%) in smokers; 0.3% (0.14% to 0.54%) in ex-smokers and 0.61% (0.013% to 1.77%) in non-smoking women with CAD.

Irrespective of the method used to measure aortic diameter the threshold size at which to diagnose AAA in women remains under debate however. Women have smaller aortas (mean diameter 1.57cm in this study) than men (mean diameter 1.79cm in NAAASP).^34^ Using a common size threshold for diagnosing AAA in women and men means that relative aortic diameter at this threshold compared to mean is greater in women than in men. Alternative approaches such as aortic size index (aortic diameter adjusted for body surface area) have been proposed.^35^ A 3cm threshold for diagnosis is approximately 1.9x normal diameter in women. Since the definition of an aneurysm is based on dilation of 50% or more above normal diameter,^36^ the data add weight to the argument that the diagnostic threshold for AAA in women be revised downwards. Identifying the relationship between the method chosen to define an AAA and future clinical events is difficult. Obviously the best threshold to choose is one that reduces the risk of future aortic rupture by identifying all cases that would go on to rupture and minimises harm from unnecessary surgical repairs. Designing a research study to define such a threshold is extremely challenging due to the low number of events when considering overall populations. It is more likely that this sort of information will be obtained from natural experiments and detailed epidemiological study of routine imaging datasets.

Screening women at high risk for AAA is unlikely to be either cost effective or clinically effective if those AAA detected have a low likelihood of repair. In the large AAAs detected in FAST, only one of three was suitable for repair, bringing into question not only the clinical bearing, but also the ethical validity of screening for a disease which may not be treatable in the majority of cases. Despite this, the clinical relevance of detecting AAA in women will require a longer period of observation. In men the potential secondary benefits of identifying AAAs through improved cardiovascular risk management have been highlighted.^37, 38^ Whether women may also benefit from improved cardiovascular risk management by AAA detection in screening is unknown. These data do show limited statin/antithrombotic use in the high-risk women who attended for screening, suggesting the potential for additional benefit here. The low prevalence of AAA and the fact that these women were easily identified through electronic clinical records suggests that there are likely to be more efficient ways to identify not only the women with AAA, but a larger group of women who would benefit from improved cardiovascular risk management.

A reduction in quality of life outcomes were identified after screening. This suggests that a screening programme for AAA in women may cause harm. The quality of life data were limited by the lack of comparable data from an unscreened population and the inability to gather pre-screening quality of life data due to the study consent model however. Addressing these potential harms of screening are an important area for future research.

The hypothesis of the study was that a targeted screening programme for women with risk factors for developing AAA would be an effective way of detecting AAA early in women and address the perceived disadvantage in a disease that remains highly morbid in the female population. However, although it remains the case that one third of AAA deaths recorded in England are in women, screening between 65 and 74 years old does not seem to be a clinically effective way of identifying AAA early in women. Longer term follow-up of our cohort of 55 diseased aortas will help to further define the natural history of AAA in women, but in a rare disease that is becoming rarer it is becoming increasingly difficult to provide good quality evidence for the best methods of screening and detection, let alone follow up and treatment. A larger study would not be of benefit to more accurately calculate prevalence in what has been established as a rare disease (in women), even in those women deemed at high risk for developing it. Health services worldwide should exercise great caution before considering implementation of targeted screening of women for AAA.

**Acknowledgements**

We would like to acknowledge the hard work of Hema Patel in administrating this study.

We also acknowledge the help of Daniel Mala and Abbey Gibbins with data entry.

**Funding**

This paper presents independent research funded by the National Institute for Health Research (NIHR) under its Research for Patient Benefit (RfPB) Programme (Grant Reference Number PB-PG-0215-36027). The views expressed are those of the author(s) and not necessarily those of the NIHR or the Department of Health and Social Care.

**Disclosures**

MJ Bown and A Saratzis are currently funded by the National Institute for Health Research (NIHR; References: NIHR202008, NIHR130075, NIHR200601, NIHR300059) and the British Hearth Foundation (BHF; Reference: RG/18/10/33842) for research relating to abdominal aortic aneurysms. A Saratzis is currently funded by Abbott Medical Ltd. (no reference) for research relating to peripheral arterial disease and receives honoraria from Philips Healthcare for research relating to abdominal aortic aneurysm treatment(s).

**References**

1. Stather PW, Sidloff DA, Rhema IA, Choke E, Bown MJ, Sayers RD. A review of current reporting of abdominal aortic aneurysm mortality and prevalence in the literature. *Eur J Vasc Endovasc Surg* 2014;**47**(3): 240-242.

2. Sidloff D, Stather P, Dattani N, Bown M, Thompson J, Sayers R, Choke E. Aneurysm global epidemiology study: public health measures can further reduce abdominal aortic aneurysm mortality. *Circulation* 2014;**129**(7): 747-753.

3. Ulug P, Powell JT, Sweeting MJ, Bown MJ, Thompson SG. Meta-analysis of the current prevalence of screen-detected abdominal aortic aneurysm in women. *Br J Surg* 2016;**103**(9): 1097-1104.

4. Thompson S, Brown L, Sweeting M, Bown M, Kim L, Glover M, Buxton M, Powell J. Systematic review and meta-analysis of the growth and rupture rates of small abdominal aortic aneurysms: implications for surveillance intervals and their cost-effectiveness. *Health technology assessment (Winchester, England)* 2013;**17**(41): 1-118.

5. Reimerink JJ, van der Laan MJ, Koelemay MJ, Balm R, Legemate DA. Systematic review and meta-analysis of population-based mortality from ruptured abdominal aortic aneurysm. *Br J Surg* 2013;**100**(11): 1405-1413.

6. Bjorck M, Bown MJ, Choke E, Earnshaw J, Florenes T, Glover M, Kay M, Laukontaus S, Lees T, Lindholt J, Powell JT, van Rij A, Svensjo S, Wanhainen A. International Update on Screening for Abdominal Aortic Aneurysms: Issues and Opportunities. *Eur J Vasc Endovasc Surg* 2014.

7. Takagi H, Ando T, Umemoto T. Abdominal Aortic Aneurysm Screening Reduces All-Cause Mortality. *Angiology* 2017: 3319717693107.

8. Glover MJ, Kim LG, Sweeting MJ, Thompson SG, Buxton MJ. Cost-effectiveness of the National Health Service Abdominal Aortic Aneurysm Screening Programme in England. *Br J Surg* 2014;**101**(8): 976-982.

9. Sweeting MJ, Masconi KL, Jones E, Ulug P, Glover MJ, Michaels JA, Bown MJ, Powell JT, Thompson SG. Analysis of clinical benefit, harms, and cost-effectiveness of screening women for abdominal aortic aneurysm. *Lancet* 2018;**392**(10146): 487-495.

10. Bown MJ, Powell JT. Part two: against the motion. Evidence does not support reducing the threshold diameter to 5 cm for elective interventions in women with abdominal aortic aneurysms. *Eur J Vasc Endovasc Surg* 2014;**48**(6): 614-618.

11. Vavra AK, Kibbe MR, Bown MJ, Powell JT. Debate: Whether evidence supports reducing the threshold diameter to 5 cm for elective interventions in women with abdominal aortic aneurysms. *J Vasc Surg* 2014;**60**(6): 1695-1701.

12. Dahl M, Frost L, Søgaard R, Klausen IC, Lorentzen V, Lindholt J. A population-based screening study for cardiovascular diseases and diabetes in Danish postmenopausal women: acceptability and prevalence. *BMC cardiovascular disorders* 2018;**18**(1): 20.

13. Kvist TV, Lindholt JS, Rasmussen LM, Søgaard R, Lambrechtsen J, Steffensen FH, Frost L, Olsen MH, Mickley H, Hallas J, Urbonaviciene G, Busk M, Egstrup K, Diederichsen ACP. The DanCavas Pilot Study of Multifaceted Screening for Subclinical Cardiovascular Disease in Men and Women Aged 65-74 Years. *Eur J Vasc Endovasc Surg* 2017;**53**(1): 123-131.

14. Scott RA, Bridgewater SG, Ashton HA. Randomized clinical trial of screening for abdominal aortic aneurysm in women. *Br J Surg* 2002;**89**(3): 283-285.

15. Svensjo S, Bjorck M, Wanhainen A. Current prevalence of abdominal aortic aneurysm in 70-year-old women. *British Journal of Surgery* 2013;**100**(3): 367-372.

16. Mureebe L, Egorova N, McKinsey JF, Kent KC. Gender trends in the repair of ruptured abdominal aortic aneurysms and outcomes. *J Vasc Surg* 2010;**51**(4 Suppl): 9s-13s.

17. Sweeting MJ, Thompson SG, Brown LC, Powell JT, Collaborators R. Meta-analysis of individual patient data to examine factors affecting growth and rupture of small abdominal aortic aneurysms. *British Journal of Surgery* 2012;**99**(5): 655-665.

18. Carter JL, Morris DR, Sherliker P, Clack R, Lam KBH, Halliday A, Clarke R, Lewington S, Bulbulia R. Sex-Specific Associations of Vascular Risk Factors With Abdominal Aortic Aneurysm: Findings From 1.5 Million Women and 0.8 Million Men in the United States and United Kingdom. *J Am Heart Assoc* 2020;**9**(4): e014748.

19. Jones GT, Hill BG, Curtis N, Kabir TD, Wong LE, Tilyard MW, Williams MJA, van Rij AM. Comparison of three targeted approaches to screening for abdominal aortic aneurysm based on cardiovascular risk. *British Journal of Surgery* 2016;**103**(9): 1139-1146.

20. Kitchener HC, Gittins M, Rivero-Arias O, Tsiachristas A, Cruickshank M, Gray A, Brabin L, Torgerson D, Crosbie EJ, Sargent A, Roberts C. A cluster randomised trial of strategies to increase cervical screening uptake at first invitation (STRATEGIC). *Health technology assessment (Winchester, England)* 2016;**20**(68): 1-138.

21. Quaife SL, Ruparel M, Dickson JL, Beeken RJ, McEwen A, Baldwin DR, Bhowmik A, Navani N, Sennett K, Duffy SW, Wardle J, Waller J, Janes SM. Lung Screen Uptake Trial (LSUT): Randomized Controlled Clinical Trial Testing Targeted Invitation Materials. *American journal of respiratory and critical care medicine* 2020;**201**(8): 965-975.

22. Wanhainen A, Verzini F, Van Herzeele I, Allaire E, Bown M, Cohnert T, Dick F, van Herwaarden J, Karkos C, Koelemay M, Kölbel T, Loftus I, Mani K, Melissano G, Powell J, Szeberin Z, Esvs Guidelines C, de Borst GJ, Chakfe N, Debus S, Hinchliffe R, Kakkos S, Koncar I, Kolh P, Lindholt JS, de Vega M, Vermassen F, Document R, Björck M, Cheng S, Dalman R, Davidovic L, Donas K, Earnshaw J, Eckstein HH, Golledge J, Haulon S, Mastracci T, Naylor R, Ricco JB, Verhagen H. Editor's Choice - European Society for Vascular Surgery (ESVS) 2019 Clinical Practice Guidelines on the Management of Abdominal Aorto-iliac Artery Aneurysms. *Eur J Vasc Endovasc Surg* 2019;**57**(1): 8-93.

23. Söderberg P, Wanhainen A, Svensjö S. Five Year Natural History of Screening Detected Sub-Aneurysms and Abdominal Aortic Aneurysms in 70 Year Old Women and Systematic Review of Repair Rate in Women. *Eur J Vasc Endovasc Surg* 2017;**53**(6): 802-809.

24. NAAASP. NHS AAA Screening Programme (online). Available: https://www.gov.uk/topic/population-screening-programmes/abdominal-aortic-aneurysm Accessed March 2021.

25. Arya R, Antonisamy B, Kumar S. Sample size estimation in prevalence studies. *Indian journal of pediatrics* 2012;**79**(11): 1482-1488.

26. Clinicaltrials.gov. FAST clinical trials registrtion page (online). Available: <https://clinicaltrials.gov/ct2/show/NCT03277781> Accessed March 2021.

27. National Institute for Health Research. FAST NIHR page (online). Available: <https://www.fundingawards.nihr.ac.uk/award/PB-PG-0215-36027> Accessed March 2021..

28. Public Health England screening programme Key Performance Indicators (online). Available: <https://www.gov.uk/government/publications/nhs-screening-programmes-kpi-reports-2019-to-2020> Accessed March 2021..

29. Public Health England AAA screening data (online) Available: <https://www.gov.uk/government/statistics/abdominal-aortic-aneurysm-screening-2018-to-2019-data> Accessed March 2021..

30. Ashton HA, Buxton MJ, Day NE, Kim LG, Marteau TM, Scott RA, Thompson SG, Walker NM. The Multicentre Aneurysm Screening Study (MASS) into the effect of abdominal aortic aneurysm screening on mortality in men: a randomised controlled trial. *Lancet* 2002;**360**(9345): 1531-1539.

31. Meecham L, Evans R, Buxton P, Allingham K, Hughes M, Rajagopalan S, Fairhead J, Asquith JR, Pherwani AD. Abdominal Aortic Aneurysm Diameters: A Study on the Discrepancy between Inner to Inner and Outer to Outer Measurements. *Eur J Vasc Endovasc Surg* 2015;**49**(1): 28-32.

32. Hartshorne TC, McCollum CN, Earnshaw JJ, Morris J, Nasim A. Ultrasound measurement of aortic diameter in a national screening programme. *Eur J Vasc Endovasc Surg* 2011;**42**(2): 195-199.

33. Hartshorne T, Naylor R, Nasim A. Use of inner versus outer wall for assessing abdominal aortic aneurysm size. *Annals of the Royal College of Surgeons of England* 2010;**92**(7): 628-629.

34. Thompson SG, Bown MJ, Glover MJ, Jones E, Masconi KL, Michaels JA, Powell JT, Ulug P, Sweeting MJ. Screening women aged 65 years or over for abdominal aortic aneurysm: a modelling study and health economic evaluation. *Health technology assessment (Winchester, England)* 2018;**22**(43): 1-142.

35. Jones GT, Sandiford P, Hill GB, Williams MJA, Khashram M, Tilyard MW, Hammond-Tooke GD, Krysa J, van Rij AM. Correcting for Body Surface Area Identifies the True Prevalence of Abdominal Aortic Aneurysm in Screened Women. *Eur J Vasc Endovasc Surg* 2019;**57**(2): 221-228.

36. Aggarwal S, Qamar A, Sharma V, Sharma A. Abdominal aortic aneurysm: A comprehensive review. *Experimental and clinical cardiology* 2011;**16**(1): 11-15.

37. Bath MF, Gokani VJ, Sidloff DA, Jones LR, Choke E, Sayers RD, Bown MJ. Systematic review of cardiovascular disease and cardiovascular death in patients with a small abdominal aortic aneurysm. *Br J Surg* 2015;**102**(8): 866-872.

38. Lederle FA. The Last (Randomized) Word on Screening for Abdominal Aortic Aneurysms. *JAMA Internal Medicine* 2016;**176**(12): 1767-1768.

**Figure legends and Tables**

**Figure 1: Attendance for AAA screening by Index of Multiple Deprivation (IMD) decile**

Error bars represent 95% confidence intervals. Lower IMD decile (lower socio-economic status) was associated with lower attendance for screening (P=0·002, R^2^ =0·7042).

**Figure 2: Distribution of aortic diameters (cm) in women aged 65-75 invited with risk factors for AAA**

Frequency of maximal aortic diameter in 5169 women who underwent scans. Range of diameters was from 0·8cm to 6·0cm with a mean aortic diameter of 1·57cm and a standard deviation of 0·27cm. Fifteen women had an aortic diameter greater than 2·9cm and were diagnosed with AAA (prevalence 0·29%). Three of these 15 women had an AAA greater the 5·5cm at screening and were referred for consideration of surgery.

**Table 1.** Demographics of women attending for screening by invitational group

|  | **All Women** | **Invited as smokers** | **Invited as ex-smokers** | **Invited as non-smokers with history of CAD** |
| --- | --- | --- | --- | --- |
| **Number consented to extended data collection** | 4613 | 1164 | 2996 | 453 |
| **Mean age (years)** | 69·6 | 69·3 | 69·6 | 70 |
| **Mean height (cm)** | 160 | 159·6 | 160·2 | 159·4 |
| **Mean weight (kg)** | 72·4 | 70·1 | 73·1 | 73·6 |
| **Mean BMI** | 28·3 | 27·5 | 28·5 | 28·9 |
| **Smoker (%)** | 726 (15·7) | 654 (56·2) | 65 (2·2) | 7 (1·5) |
| **Ex-smoker (%)** | 3297 (71·5) | 473 (40·6) | 2719 (90·8) | 105 (23·2) |
| **e-cigarette user (%)** | 248 (5·4) | 143 (12·3) | 101 (3·4) | 4 (0·9) |
| **Diabetes (%)** | 452 (9·8) | 122 (10·5) | 274 (9·1) | 56 (12·4) |
| **Stroke (%)** | 207 (4·5) | 70 (6·0) | 123 (4·1) | 14 (3·1) |
| **MI (%)** | 205 (4·4) | 43 (3·7) | 90 (3·0) | 72 (15·9) |
| **CABG (%)** | 40 (0·9) | 14 (1·2) | 10 (0·3) | 16 (3·5) |
| **Coronary Angiogram (%)** | 544 (11·8) | 152 (13·1) | 245 (8·2) | 147 (32·5) |
| **Coronary stents (%)** | 171 (3·7) | 34 (2·9) | 71 (2·4) | 66 (14·6) |
| **PAD (%)** | 68 (1·5) | 40 (3·4) | 26 (0·9) | 2 (0·4) |
| **Hypertension (%)** | 2013 (43·6) | 468 (40·2) | 1287 (43·0) | 258 (57·0) |
| **Antihypertensives (%)** | 1955 (42·4) | 473 (40·6) | 1233 (41·2) | 249 (55·0) |
| **Hypercholesterolaemia (%)** | 2010 (43·6) | 492 (42·3) | 1257 (42·0) | 261 (57·6) |
| **Aspirin (%)** | 537 (11·6) | 133 (11·4) | 273 (9·1) | 131 (28·9) |
| **Clopidogrel (%)** | 167 (3·6) | 61 (5·2) | 92 (3·1) | 14 (3·1) |
| **Warfarin (%)** | 107 (2·3) | 29 (2·5) | 62 (2·1) | 16 (3·5) |
| **Statin (%)** | 1763 (38·2) | 469 (40·3) | 1060 (35·4) | 234 (51·7) |
| **Anticoagulant (%)** | 117 (2·5) | 25 (2·1) | 77 (2·6) | 15 (3·3) |
| **No medication (%)** | 2160 (46·8) | 497 (42·7) | 1533 (51·2) | 130 (28·7) |
| **Family history of AAA (%)** | 325 (7·0) | 83 (7·1) | 217 (7·2) | 25 (5·5) |
| **Mother (%)** | 103 (2·2) | 30 (2·6) | 62 (2·1) | 11 (2·4) |
| **Father (%)** | 144 (3·1) | 25 (2·1) | 108 (3·6) | 11 (2·4) |
| **Brother (%)** | 65 (1·4) | 18 (1·5) | 44 (1·5) | 3 (0·7) |
| **Sister (%)** | 20 (0·4) | 10 (0·9) | 10 (0·3) | 0 (0·0) |
| **Previous USS/CT/MR (%)** | 2037 (44·2) | 605 (52·0) | 1192 (39·8) | 240 (53·0) |

Collated data of all women who consented to extended data collection, both in total and by invitation group. Percentages (shown in parentheses) are the percentage of women with that finding within each invitational group.

**Table 2:** Prevalence (95% CI) of sub-aneurysmal aortic dilatation/AAA by invitation group and directly recorded risk factors for AAA

| **Invitation group/risk factor** | **Normal aorta**  **(aortic diameter <2.5cm)** | **Sub-aneurysmal aortic dilatation**  **(aortic diameter 2.5cm-2.9cm)** | **AAA**  **(aortic diameter ≥3.0cm** |
| --- | --- | --- | --- |
| All Women | 98.96 (98.63-99.21) | 0.75 (0.54-1.04) | 0.29 (0.17-0.49) |
| Invited as smoker | 97.78 (96.79-98.48) | 1.53 (0.96-2.40) | 0.69 (0.34-1.36) |
| Invited as ex-smoker | 99.35 (99.00-99.58) | 0.47 (0.28-0.78) | 0.18 (0.07-4.10) |
| Invited as non-smoker with CAD* | 99.39 (98.22-99.79) | 0.61 (0.21-1.78) | 0.00 (0.00-0.77) |
| Actual smoker | 96.96 (95.36-98.04) | 2.21 (2.31-3.64) | 0.83 (0.34-1.89) |
| Actual ex-smoker | 99.18 (98.79-99.45) | 0.58 (0.36-0.92) | 0.24 (0.11-0.49) |
| Actual non-smoker with CAD | 100.00 (96.67-100.00) | 0.00 (0.00-2.67) | 0.00 (0.00-2.67) |
| Actual current smoker with CAD | 97.62 (91.73-99.35) | 1.19 (0.21-6.44) | 1.19 (0.21-6.44) |
| Actual ex-smoker with CAD | 97.76 (95.46-98.95) | 1.96 (0.86-4.17) | 0.84 (0.29-2.44) |

*Coronary Artery Disease.
