# Supplementary material for: Ultrasound screening for abdominal aortic aneurysm in high-risk women
Source: Br J Surg. 2021 Aug 9;108(10):1192–8. doi: 10.1093/bjs/znab220 (PMC8545265; doi:10.1093/bjs/znab220)
Supplement: znab220_Supplementary_Data [file znab220_supplementary_data.zip › Supplementary information.docx]

**Ultrasound screening for abdominal aortic aneurysm in high-risk women. The Female Aneurysm screening STudy (FAST) Supplementary Information, Tables and Figures.**

**Supplementary methods:**

**Screening protocol:**

The screening ultrasound was completed by one of three trained clinical scientists using a Samsung HM70A device (Samsung, South Korea) with a CA1-7AD curvi-linear probe, a device approved by the NHS AAA Screening Programme (NAAASP) for AAA screening in men. Those conducting screening had been trained and had screened at least 100 women under the supervision of the local NAAASP team, as per NAAASP Standard Operating Procedures (SOPs),^(1)^ and had passed a relevant clinical assessment by the lead vascular ultrasound scientist in Leicestershire (author TH). Two images of the aorta were captured at the area of maximal diameter in axial and sagittal planes perpendicular to the aorta after screening the entire visible aorta to the aortic bifurcation. Measurements were taken from inner edge to inner edge and were recorded to one decimal place, as per NAAASP screening protocols.^(1)^

**Supplementary results:**

**Quality of Life analyses**

Overall, 993 women completed both an initial EQ-5D-5L and a repeat questionnaire at six months; 990 completed Visual Analogue Scores (VAS) on both forms, and 979 adequately completed the questionnaire to allow for conversion to index values using the crosswalk method. Due to the low prevalence of abnormal aortic findings (AAA or sub-aneurysm) there was inadequate data for quality of life analysis by screening outcome sub-groups. Five women with AAA completed baseline EQ-5D and only 3 also completed the 6-month EQ-5D. For the sub-aneurysm group 12 completed the baseline EQ-5D and 5 both baseline and 6-month EQ5D. Using the Pareto Classification of Health Change, between the initial and six-month post screening timepoints EQ-5D health states were the same or better in 45·3% of respondents, mixed in 17·2%, and worse in 37·5%.^(2)^ Crosswalk index values were calculated using the method described by Van Hout et al.^(3)^ The average crosswalk index values decreased significantly by 0·04 (95% CI: 0·02 - 0·06) from 0·797 (95% CI: 0·784 - 0·81) pre-test to 0·755 (95% CI: 0·74 - 0·77) post-test (p=0·001) (Supplementary Table 2). The median (IQR) VAS also decreased significantly from 85 (70 to 95) at baseline to 81·5 (70 to 91·5) at follow up (p=0·0001). When analysed by response dimension there was a significant decrease in patient reported outcomes (PRO) in all except the mobility domain between the baseline and follow up respondents using the chi-squared test. When compared with the national data published by the EuroQol group for women aged 65 to 74 in the UK, the dichotomised baseline data did not differ significantly from the population norms reported.^(4)^ When follow up data was compared there were significant reductions in the self-care, usual activities and pain/discomfort dimensions. These results demonstrate a significant reduction in PRO (Supplementary Figure 5 and Supplementary Tables 3, and 4).

**Quality assurance of screening**

The main indicators of screening quality used by NAAASP are a) the number of screening encounters where the aorta can not be visualised on the first scan (“non-visualisation”) and b) the number of overall non-conclusive screening episodes due to an inability to image the aorta.^(5)^ Fifty women attending for screening had “non-visualisation” screening encounters on their first visit (0·96%); less than the figure of 1·2% observed in men.^(5, 6)^ 35 of these women went on to have normal scans at a second appointment. In the remaining 15 women (0·29%), of the total 5,190 women who underwent a screening ultrasound, a conclusive screening result was never obtained due to inability to successful image the aorta using ultrasound. This is below the NAAASP England average of 0·37%.^(5, 6)^ The average body mass index in the non-visualisation group compared to that in the group with successful first scans was significantly higher (39 kg/m2, SD 8·25 vs. 28 kg/m2, SD: 6, p=0·0001).

**Accuracy of primary care records for AAA risk factors**

Actual smoking status in women attending for screening was compared with that used as the basis of invitation for screening. Overall, 56·2% of those invited as current smokers based on their GP records stated they were still currently smoking at the time of screening. The accuracy of primary care records regarding ex-smokers and non-smokers was higher at 90·8% and 75·3%, respectively. When combining the two cohorts into an ‘ever-smokers’ group the accuracy of coding was 94·1% (Supplementary Table 5).

**Supplementary references:**

1. Public Health England. NHS AAA Screening Programme Standard Operating Procedures (web). Available: [https://www.gov.uk/government/publications/aaa-screening-standard-operating-procedures. Accessed 5/10/2020](https://www.gov.uk/government/publications/aaa-screening-standard-operating-procedures.%20Accessed%205/10/2020)
2. Devlin NJ, Parkin D, Browne J. Patient-reported outcome measures in the NHS: new methods for analysing and reporting EQ-5D data. Health economics. 2010;19(8):886—905.
3. Van Hout B, Janssen M, Feng Y-S, Kohlmann T, Busschbach J, Golicki D, et al. Interim scoring for the EQ-5D-5L: mapping the EQ-5D-5L to EQ-5D-3L value sets. Value in health. 2012;15(5):708—15.
4. Janssen B, Cabases J, Szende A. Self-reported population health: an international perspective based on EQ-5D: Springer Netherlands; 2014.
5. Public Health England. Abdominal aortic aneurysm screening programme standards valid for data collected from 1 April 2020 (web). Available: https://www.gov.uk/government/publications/aaa-screening-quality-standards-and-service-objectives/abdominal-aortic-aneurysm-screening-programme-standards-valid-for-data-collected-from-1-april-2020. Accessed 25/10/2020.
6. Public Health England. AAA screening standards: data report 1 April 2018 to 31 March 2019 (web). Available: https://www.gov.uk/government/publications/abdominal-aortic-aneurysm-screening-2018-to-2019-data/aaa-screening-standards-data-report-1-april-2018-to-31-march-2019. Accessed 25/10/2020.24

**Supplementary figures and tables**


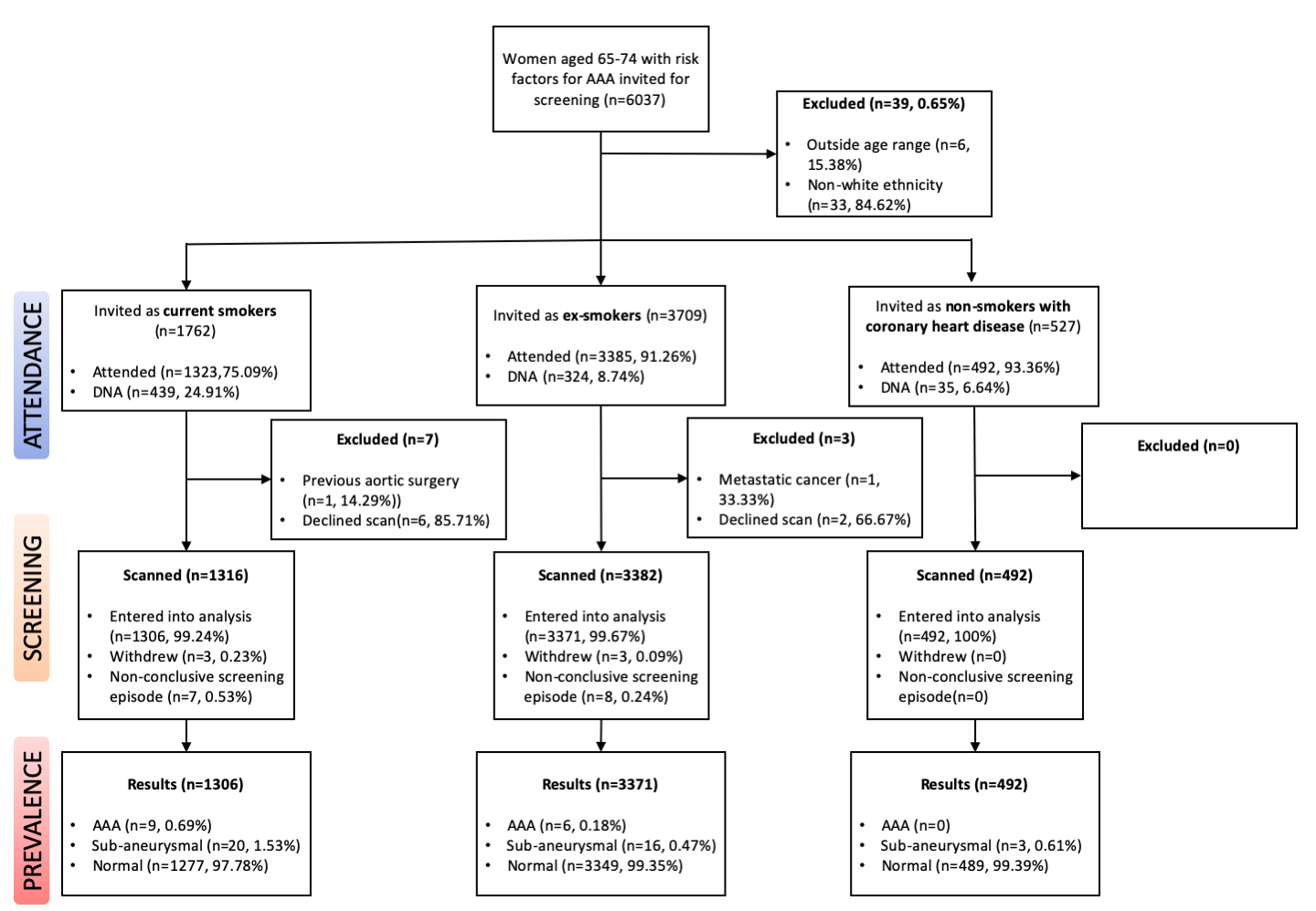
**Supplementary Figure 1: Study flow diagram -** the number of participants at each stage of the study, including the number and reasons for exclusion.

**Supplementary Figure 2. Attendance for AAA screening by criteria for invitation and method of invitation**


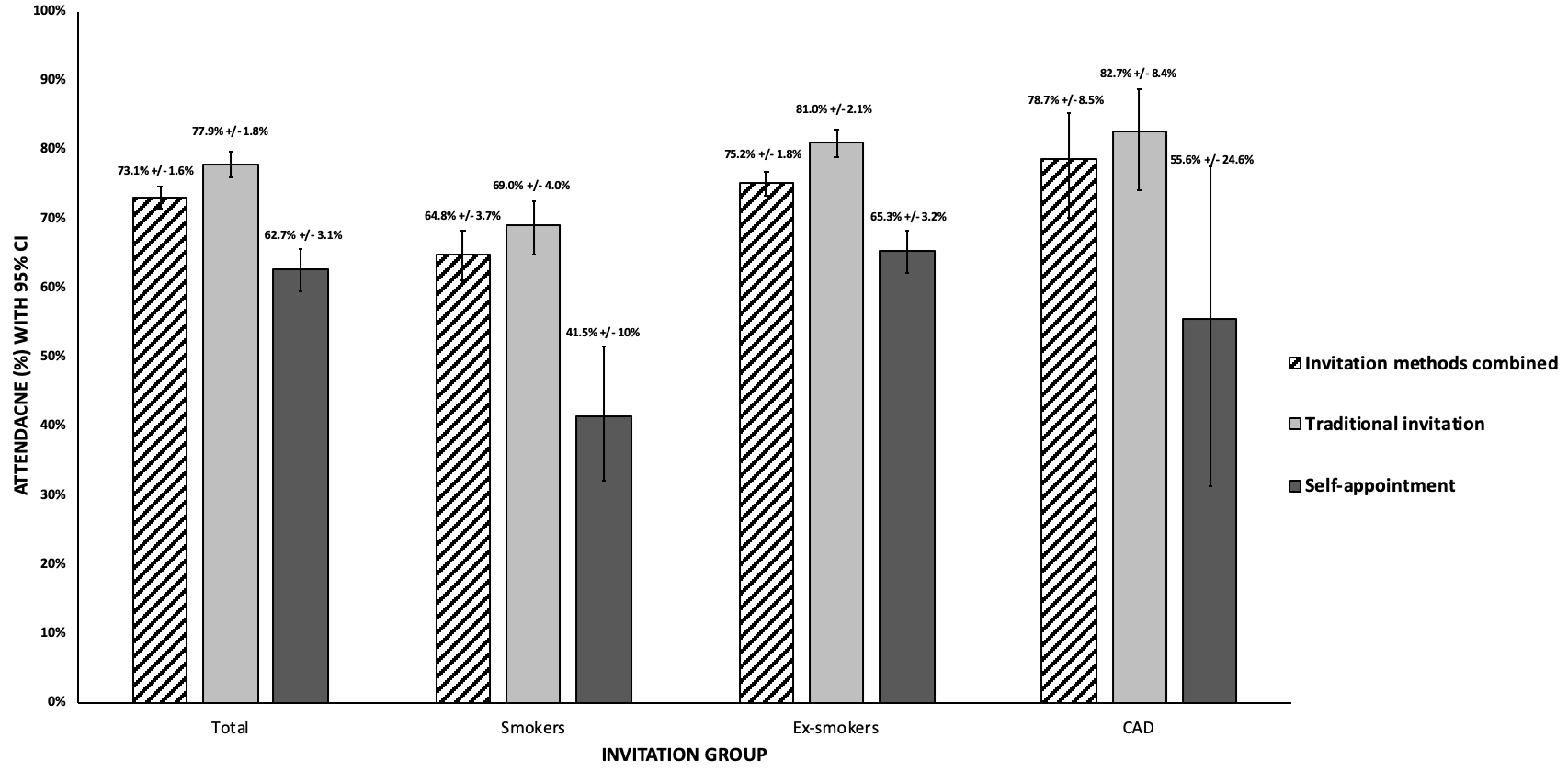


Percentage attendance by invitation method and group. Error bars show 95% confidence intervals. The standard NAAASP method of invitation was considered the ‘traditional invitation’. An alternative ‘self-invitation’ process was used in one practice where women were asked to arrange their own appointment for screening if they wished to be screened. Attendance was significantly better in the traditional invitation than the self-appointment group (p<0·0002). Smokers were significantly less likely to attend than ex-smokers of non-smokers with CAD (p<0·05).

**Supplementary Figure 3: Attendance for AAA screening by year of age**

Error bars represent 95% confidence intervals. There was no correlation between age and attendance for screening (P = 0·96, R^2^ = 0·0104).

**Supplementary Figure 4. Clinic utilisation by invitation method**


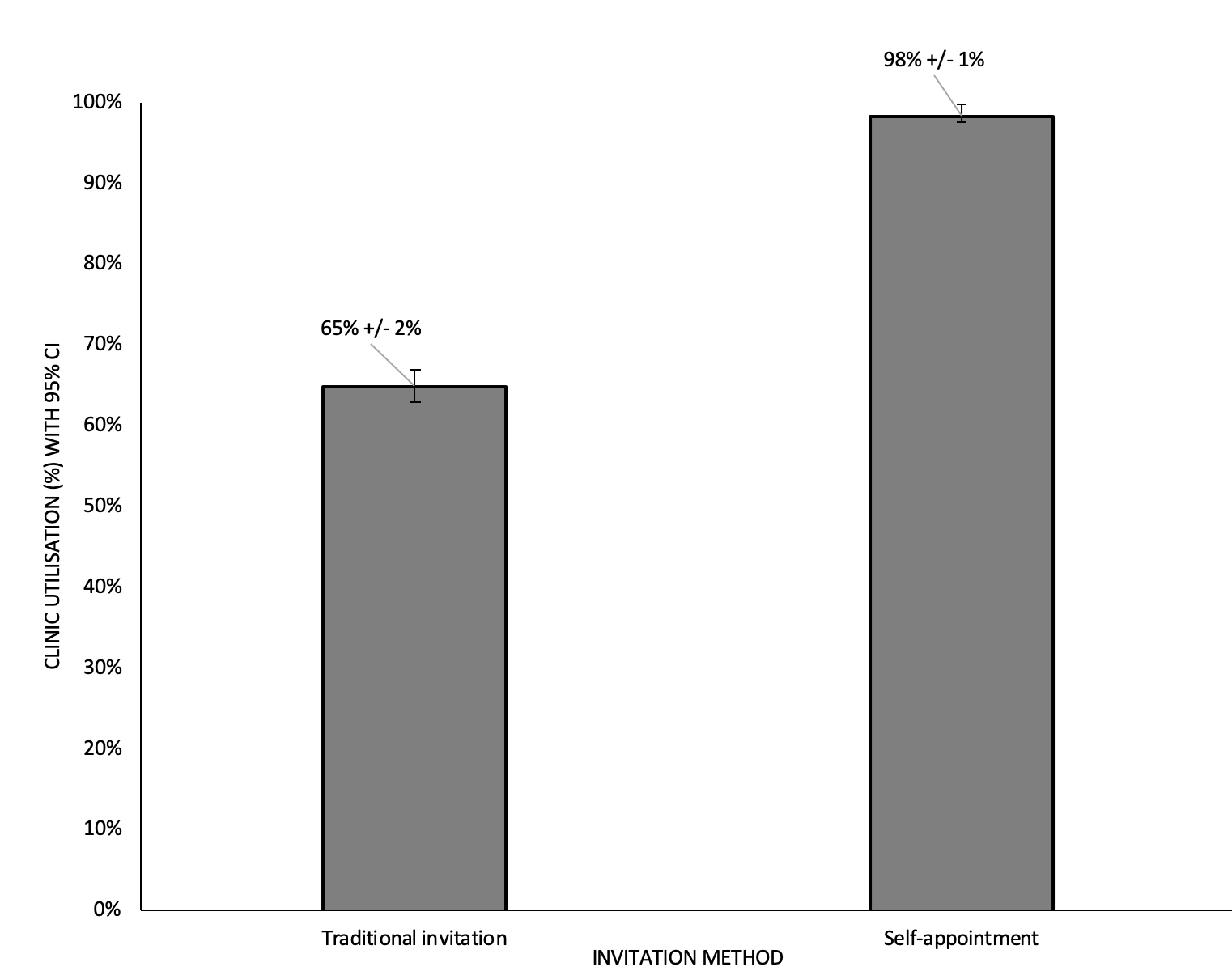


Error bars show 95% confidence intervals. Clinic utilisation was higher using a self-appointment method (P<0·0002).

**Supplementary Figure 5.** **Mean EQ-5D-5L Index values from initial screening and six month follow up**


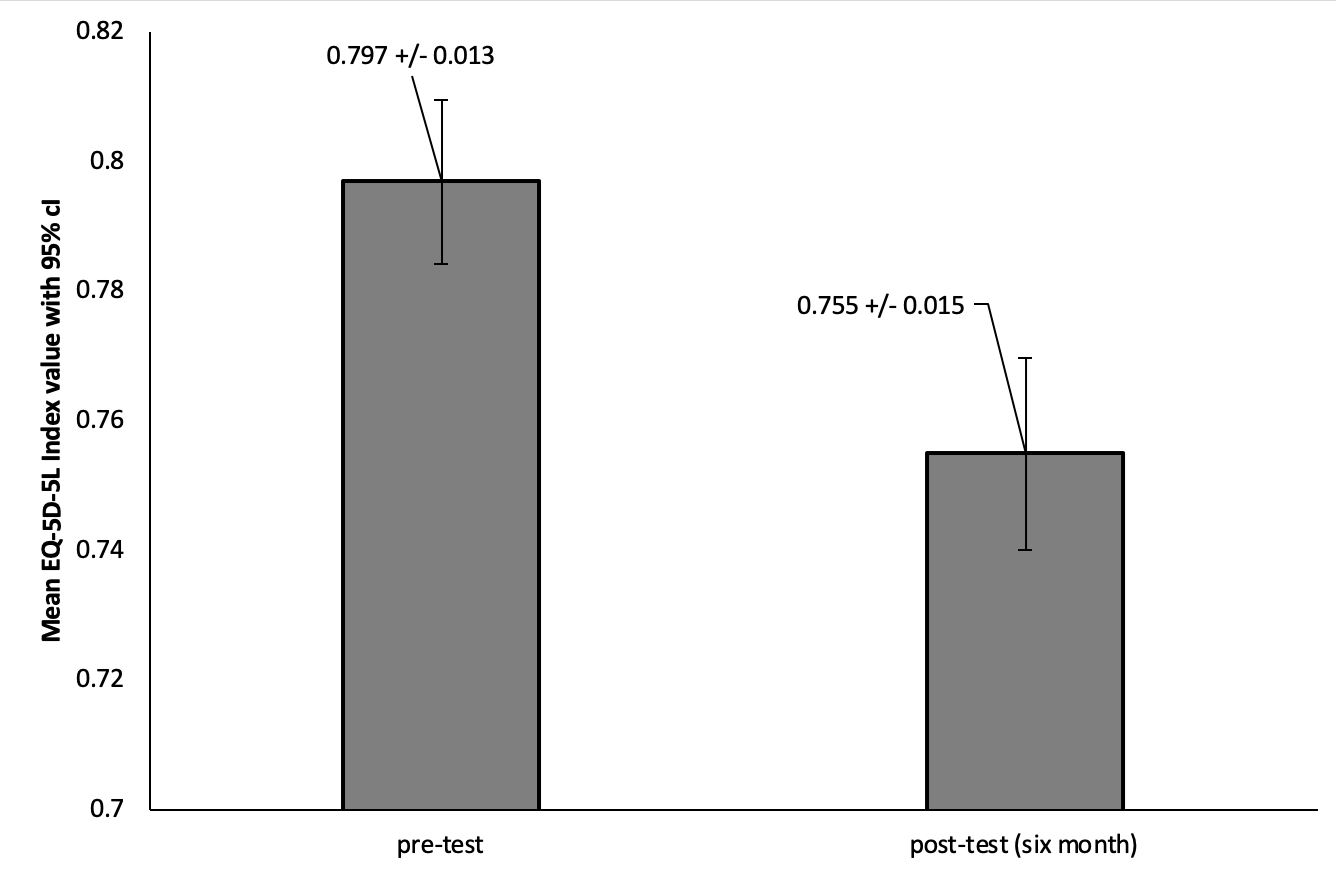


Error bars show 95% confidence intervals. Crosswalk index values decreased significantly (p=0·001) in the six months from the first test.

**Supplementary Table 1. Comorbidities in women with normal aortic diameter, subaneurymal aortic dilatation and AAA.**

|  | **Normal aorta**  **(aortic diameter <2.5cm)** | **Sub-aneurysmal aortic dilatation**  **(aortic diameter 2.5cm-2.9cm)** | **AAA**  **(aortic diameter ≥3.0cm** |
| --- | --- | --- | --- |
| **Number consented to extended data collection** | 4559 | 35 | 14 |
| **Mean age (years)** | 69.57 | 69.43 | 71.00 |
| **Mean height (cm)** | 159.98 | 161.31 | 162.69 |
| **Mean weight (kg)** | 72.37 | 73.30 | 75.01 |
| **Mean BMI** | 28.28 | 28.25 | 28.43 |
| **Smoker (%)** | 702 (15.4) | 16 (45.71) | 6 (42.86) |
| **Ex-smoker (%)** | 3267 (71.66) | 19 (54.29) | 8 (57.14) |
| **e-cigarette user (%)** | 239 (5.24) | 8 (22.86) | 1 (7.14) |
| **Diabetes (%)** | 442 (9.7) | 4 (11.43) | 4 (28.57) |
| **Stroke (%)** | 203 (4.45) | 2 (5.71) | 2 (14.29) |
| **MI (%)** | 199 (4.36) | 3 (8.57) | 3 (21.43) |
| **CABG (%)** | 40 (0.88) | 0 (0) | 0 (0) |
| **Coronary Angiogram (%)** | 535 (11.74) | 7 (20.00) | 2 (14.29) |
| **Coronary stents (%)** | 166 (3.64) | 4 (11.43) | 1 (7.14) |
| **PAD (%)** | 66 (1.45) | 1 (2.86) | 1 (7.14) |
| **Hypertension (%)** | 1985 (43.54) | 18 (51.43) | 7 (50.00) |
| **Antihypertensives (%)** | 1926 (42.25) | 18 (51.43) | 8 (57.14) |
| **Hypercholesterolaemia (%)** | 1982 (43.47) | 19 (54.29) | 7 (50.00) |
| **Aspirin (%)** | 530 (11.63) | 5 (14.29) | 2 (14.29) |
| **Clopidogrel (%)** | 165 (3.62) | 2 (5.71) | 0 (0) |
| **Warfarin (%)** | 103 (2.26) | 3 (8.57) | 1 (7.14) |
| **Statin (%)** | 1742 (38.21) | 14 (40) | 6 (42.86) |
| **Anticoagulant (%)** | 114 (2.50) | 1 (2.86) | 1 (7.14) |
| **No medication (%)** | 2135 (46.83) | 16 (45.71) | 6 (42.86) |
| **Family history of AAA (%)** | 316 (6.93) | 6 (17.14) | 3 (21.43) |
| **Mother (%)** | *97 (30.70)* | *4 (66.67)* | *2 (66.67)* |
| **Father (%)** | *142 (44.94)* | *1 (16.67)* | *1 (33.33)* |
| **Brother (%)** | *65 (20.57)* | *0 (0)* | *0 (0)* |
| **Sister (%)** | *20 (6.33)* | *0 (0)* | *0 (0)* |
| **Previous USS/CT/MR (%)** | *1 (0.32)* | *1 (16.67)* | *0 (0)* |

**Supplementary Table 2. Baseline and Follow up EQ-5D-5L scores**

| **Domain** | **Baseline n (%)** | **Follow up n (%)** | **P Value** |
| --- | --- | --- | --- |
|  |  |  |  |
| Mobility |  |  |  |
| **1 (no problems)** | 594 (59·8) | 556 (56·3) | **NS** |
| **2 (slight problems)** | 184 (18·5) | 205 (20·8) |  |
| **3 (moderate problems)** | 133 (13·3) | 138 (14·0) |  |
| **4 (severe problems)** | 80 (8·1) | 84 (8·5) |  |
| **5 (unable to/extreme pain/anxiety)** | 2 (0·2) | 4 (0·41) |  |
| **9 (unfilled)** | 0 | 6 |  |
| **Total respondents** | 993 | 987 |  |
| Self Care |  |  |  |
| **1 (no problems)** | 909 (91·5) | 839 (84·7) | **< 0·001** |
| **2 (slight problems)** | 44 (4·4) | 83 (8·4) |  |
| **3 (moderate problems)** | 33 (3·3) | 50 (5·1) |  |
| **4 (severe problems)** | 5 (0·5) | 14 (1·4) |  |
| **5 (unable to/extreme pain/anxiety)** | 2 (0·2) | 4 (0·4) |  |
| **9 (unfilled)** | 0 | 3 |  |
| **Total respondents** | 993 | 990 |  |
| Usual Activities |  |  |  |
| **1 (no problems)** | 717 (72·2) | 573 (58·2) | **< 0·001** |
| **2 (slight problems)** | 152 (15·3) | 217 (22·0) |  |
| **3 (moderate problems)** | 83 (8·4) | 129 (13·1) |  |
| **4 (severe problems)** | 36 (3·6) | 52 (5·3) |  |
| **5 (unable to/extreme pain/anxiety)** | 5 (0·5) | 14 (1·4) |  |
| **9 (unfilled)** | 0 | 8 |  |
| **Total respondents** | 993 | 985 |  |
| Pain/Discomfort |  |  |  |
| **1 (no problems)** | 462 (46·5) | 344 (34·7) | **< 0·001** |
| **2 (slight problems)** | 271 (27·3) | 362 (36·6) |  |
| **3 (moderate problems)** | 187 (18·8) | 196 (19·8) |  |
| **4 (severe problems)** | 65 (6·5) | 75 (7·6) |  |
| **5 (unable to/extreme pain/anxiety)** | 8 (0·8) | 13 (1·3) |  |
| **9 (unfilled)** | 0 | 3 |  |
| **Total respondents** | 993 | 990 |  |
| Anxiety/ depression |  |  |  |
| **1 (no problems)** | 678 (68·3) | 628 (63·5) | **0·03** |
| **2 (slight problems)** | 201 (20·2) | 244 (24·7) |  |
| **3 (moderate problems)** | 97 (9·8) | 87 (8·8) |  |
| **4 (severe problems)** | 12 (1·2) | 23 (2·3) |  |
| **5 (unable to/extreme pain/anxiety)** | 5 (0·5) | 7 (0·7) |  |
| **9 (unfilled)** | 0 | 4 |  |
| **Total respondents** | 993 | 989 |  |

Number of respondents reporting outcome by EQ-5D-5L domain in both the initial and follow up questionnaire. Analysed by chi-squared test, the quality of life domains saw a significant decrease in all by the mobility domain with p values displayed.

**Supplementary Table 3.** **Baseline dichotomised data compared with EQ-5D index data for UK women aged 65 to 74**

|  | | | |
| --- | --- | --- | --- |
|  |  |  |  |
| **Domain** | **Baseline n(%)** | **Index data n(%)** | **P Value** |
|  |  |  |  |
| Mobility |  |  |  |
| **No problems** | 594 (59·8) | 164 (63·1) | **NS** |
| **Any problems** | 399 (40·2) | 96 (36·9) |  |
| Self Care |  |  |  |
| **No problems** | 909 (91·5) | 244 (93·8) | **NS** |
| **Any problems** | 84 (8·5) | 16 (6·2) |  |
| Usual Activities |  |  |  |
| **No problems** | 717 (72·2) | 189 (72·7) | **NS** |
| **Any problems** | 276 (27·8) | 71 (27·3) |  |
| Pain/Discomfort |  |  |  |
| **No problems** | 462 (46·5) | 126 (48·5) | **NS** |
| **Any problems** | 531 (53·5) | 134 (51·5) |  |
| Anxiety/ depression |  |  |  |
| **No problems** | 678 (68·3) | 180 (69·2) | **NS** |
| **Any problems** | 315 (31·7) | 80 (30·8) |  |

Dichotomised quality of life data from EQ-5D-5L baseline and follow up questionnaires, showing no significant difference with the standardised EuroQoL dataset for UK women in the same age group.

**Supplementary Table 4.** **Follow up dichotomised data compared with EQ-5D index data for UK women aged 65 to 74**

|  | | | |
| --- | --- | --- | --- |
|  |  |  |  |
| **Domain** | **Follow up n(%)** | **Index data up n(%)** | **P Value** |
|  |  |  |  |
| Mobility |  |  |  |
| **No problems** | 556 (56·3) | 164 (63·1) | **NS** |
| **Any problems** | 431 (43·7) | 96 (36·9) |  |
| Self Care |  |  |  |
| **No problems** | 839 (84·7) | 244 (93·8) | **< 0·001** |
| **Any problems** | 151 (15·3) | 16 (6·2) |  |
| Usual Activities |  |  |  |
| **No problems** | 573 (58·2) | 189 (72·7) | **<0·001** |
| **Any problems** | 412 (41·8) | 71 (27·3) |  |
| Pain/Discomfort |  |  |  |
| **No problems** | 344 (34·7) | 126 (48·5) | **<0·001** |
| **Any problems** | 646 (65·3) | 134 (51·5) |  |
| Anxiety/ depression |  |  |  |
| **No problems** | 628 (63·5) | 180 (69·2) | **NS** |
| **Any problems** | 361 (36·5) | 80 (30·8) |  |

Dichotomised quality of life data from EQ-5D-5L follow up questionnaire (6 months) showing significant more respondents with “any problem” in all but the mobility domain when compared to the standardised EuroQoL dataset for UK women in the same age group.

**Supplementary Table 5. Accuracy of General Practitioner (GP) record coding**

| **Primary care clinical system smoking status indicator** | **Patient reported current smoker** | **Patient reported ex-Smoker** | **Patient reported non-smoker** | **Total** |
| --- | --- | --- | --- | --- |
| Current smoker | 654 (56·2) | 473 (40·7) | 36 (3·1) | 1163 |
| Ex-smoker | 65 (2·2) | 2719 (90·8) | 209 (7·0) | 2993 |
| Non-smoker | 7 (1·5) | 105 (23·2) | 341 (75·3) | 453 |
| Current or ex-smokers | 719 (17·3) | 3192 (76·8) | 245 (5·9) | 4156 |

Number and percentage of women reporting smoking status at the time of screening by smoking status recorded in primary care clinical system (basis of invitation for screening). Percentages (shown in parentheses) are the percentage within each primary care clinical system smoking status group (rows). Accuracy of smoking status recorded by primary care databases is compared to the smoking status reported by the patient on questioning. Although not representative for indicating smoking status in current smokers (only 56·2% correctly recorded), when combined into an ‘ever-smokers’ group (current and ex-smokers) GP clinical system coding was more representative of smoking status (94·1%).

STROBE Statement—Checklist of items that should be included in reports of ***cohort studies***

**Ultrasound screening for abdominal aortic aneurysm in high-risk women. The Female Aneurysm screening STudy (FAST)**

|  | Item No | Recommendation |
| --- | --- | --- |
| **Title and abstract** | 1 | (*a*) Indicate the study’s design with a commonly used term in the title or the abstract |
|  |  | (*b*) Provide in the abstract an informative and balanced summary of what was done and what was found  Included in the manuscript |
| Introduction | | |
| Background/rationale | 2 | Explain the scientific background and rationale for the investigation being reported  Included in the manuscript |
| Objectives | 3 | State specific objectives, including any prespecified hypotheses  Included in the manuscript |
| Methods | | |
| Study design | 4 | Present key elements of study design early in the paper  Included in the manuscript |
| Setting | 5 | Describe the setting, locations, and relevant dates, including periods of recruitment, exposure, follow-up, and data collection  Included in the manuscript |
| Participants | 6 | (*a*) Give the eligibility criteria, and the sources and methods of selection of participants. Describe methods of follow-up  Included in the manuscript |
|  |  | (*b*) For matched studies, give matching criteria and number of exposed and unexposed  Included in the manuscript |
| Variables | 7 | Clearly define all outcomes, exposures, predictors, potential confounders, and effect modifiers. Give diagnostic criteria, if applicable  Included in the manuscript |
| Data sources/ measurement | 8* | For each variable of interest, give sources of data and details of methods of assessment (measurement). Describe comparability of assessment methods if there is more than one group  Included in the manuscript |
| Bias | 9 | Describe any efforts to address potential sources of bias  Included in the manuscript |
| Study size | 10 | Explain how the study size was arrived at  Included in the manuscript |
| Quantitative variables | 11 | Explain how quantitative variables were handled in the analyses. If applicable, describe which groupings were chosen and why  Included in the manuscript |
| Statistical methods | 12 | (*a*) Describe all statistical methods, including those used to control for confounding |
|  |  | (*b*) Describe any methods used to examine subgroups and interactions |
|  |  | (*c*) Explain how missing data were addressed |
|  |  | (*d*) If applicable, explain how loss to follow-up was addressed |
|  |  | (*e*) Describe any sensitivity analyses  All the above included in the manuscript |
| Results | | |
| Participants | 13* | (a) Report numbers of individuals at each stage of study—eg numbers potentially eligible, examined for eligibility, confirmed eligible, included in the study, completing follow-up, and analysed |
|  |  | (b) Give reasons for non-participation at each stage |
|  |  | (c) Consider use of a flow diagram  All the above included in the manuscript |
| Descriptive data | 14* | (a) Give characteristics of study participants (eg demographic, clinical, social) and information on exposures and potential confounders |
|  |  | (b) Indicate number of participants with missing data for each variable of interest |
|  |  | (c) Summarise follow-up time (eg, average and total amount)  All the above included in the manuscript |
| Outcome data | 15* | Report numbers of outcome events or summary measures over time |
| Main results | 16 | (*a*) Give unadjusted estimates and, if applicable, confounder-adjusted estimates and their precision (eg, 95% confidence interval). Make clear which confounders were adjusted for and why they were included |
|  |  | (*b*) Report category boundaries when continuous variables were categorized |
|  |  | (*c*) If relevant, consider translating estimates of relative risk into absolute risk for a meaningful time period  All the above included in the manuscript |
| Other analyses | 17 | Report other analyses done—eg analyses of subgroups and interactions, and sensitivity analyses  Included in the manuscript |
| Discussion | | |
| Key results | 18 | Summarise key results with reference to study objectives  Included in the manuscript |
| Limitations | 19 | Discuss limitations of the study, taking into account sources of potential bias or imprecision. Discuss both direction and magnitude of any potential bias  Included in the manuscript |
| Interpretation | 20 | Give a cautious overall interpretation of results considering objectives, limitations, multiplicity of analyses, results from similar studies, and other relevant evidence  Included in the manuscript |
| Generalisability | 21 | Discuss the generalisability (external validity) of the study results  Included in the manuscript |
| Other information | | |
| Funding | 22 | Give the source of funding and the role of the funders for the present study and, if applicable, for the original study on which the present article is based  Included in the manuscript |

*Give information separately for exposed and unexposed groups.
